# Movement behavior of swordfish provisions connectivity between the temperate and tropical southwest Pacific Ocean

**DOI:** 10.1038/s41598-023-38744-z

**Published:** 2023-07-21

**Authors:** Sean R. Tracey, Barrett W. Wolfe, Klaas Hartmann, Julian Pepperell, Sam M. Williams

**Affiliations:** 1 Institute for Marine and Antarctic Studies, University of Tasmania, Private Bag 49, Hobart, TAS 7001 Australia; 2 Pepperell Research and Consulting Pty Ltd, P.O. Box 1475, Noosaville DC, QLD 4566 Australia; 3 School of Biomedical Sciences, The University of Queensland, St Lucia, QLD 4072 Australia

**Keywords:** Marine biology, Animal migration, Behavioural ecology, Biooceanography

## Abstract

Swordfish (*Xiphias gladius*) are a widely distributed (45°N–45°S) large pelagic fish targeted by fisheries worldwide. Swordfish that occur at high latitudes tend to disproportionately be large adults, so their movements have implications for population dynamics and fisheries management. In the southwest Pacific, little is known about this subset of the stock and existing evidence suggests limited movement from the subtropics into cooler high latitude waters. Here, we capitalize on the recent emergence of a recreational swordfish fishery off temperate southeast Australia to characterize movements of swordfish caught in the fishery with pop-up satellite archival transmitting tags. Data were recovered from tags deployed for 56–250 days on 11 swordfish (50–350 kg) tagged between 38 and 43°S in the western Tasman Sea. Five swordfish entered the Coral Sea (< 30°S), with four reaching north to 11–24°S, up to 3275 km away from location of capture. Behavior modelling suggests these four individuals rapidly transited north until encountering 23–27 °C water, at which point they lingered in the area for several months, consistent with spawning-related partial migration. One migrating swordfish still carrying a tag after the spawning season returned to ~ 120 km of its release location, suggesting site fidelity. Movements toward the central south Pacific were confined to two individuals crossing 165°E. Swordfish predominantly underwent normal diel vertical migration, descending into the mesopelagic zone at dawn (median daytime depth 494.9 m, 95% CI 460.4–529.5 m). Light attenuation predicted daytime depth, with swordfish rising by up to 195 m in turbid water. At night, swordfish were deeper during the full moon, median night-time depth 45.8 m (37.8–55.5) m versus 18.0 m (14.9–21.8) m at new moon. Modelling fine-scale (10 min^−1^) swordfish depth revealed dynamic effects of moon phase varying predictably across time of night with implications for fisheries interactions. Studying highly migratory fishes near distribution limits allows characterization of the full range of movement phenotypes within a population, a key consideration for important fish stocks in changing oceans.

## Introduction

The swordfish (*Xiphias gladius*) is a highly migratory, epi- and mesopelagic predator distributed throughout the world’s oceans from 45°N to 45°S^1^. The species is important ecologically and commercially, with annual global catches of over 100,000 metric tons since 2000^2^. Swordfish are targeted along both the west and east coasts of Australia. While those targeted adjacent to the west coast of Australia are considered to be part of a single Indian Ocean biological stock^3,4^, Pacific Ocean swordfish population structure to the east is more complex. Working hypotheses include two, three, and four stocks^5^. For management purposes, however, the fish adjacent to the east coast of Australia are considered part of a southwest Pacific Ocean stock, managed by the Western and Central Pacific Fisheries Commission (WCPFC). The most recently available data indicated a southern hemisphere WCPFC harvest of 5,516 t in 2020, including 611 t harvested by Australian vessels endorsed in the Eastern Tuna and Billfish Fishery, and the fishery is currently considered ‘not overfished’ and ‘not subject to overfishing’^6^. While many aspects of swordfish movement behavior remain unresolved available evidence from the southwest Pacific suggests that the species’ population structure and susceptibility to fishing pressure may be influenced by their movement across the region^7–9^. As such, characterizing movements and habitat use of swordfish both within the southwest Pacific and between adjoining regions remains a longstanding research priority to support fisheries management^7,10,11^.

**Figure 1:**
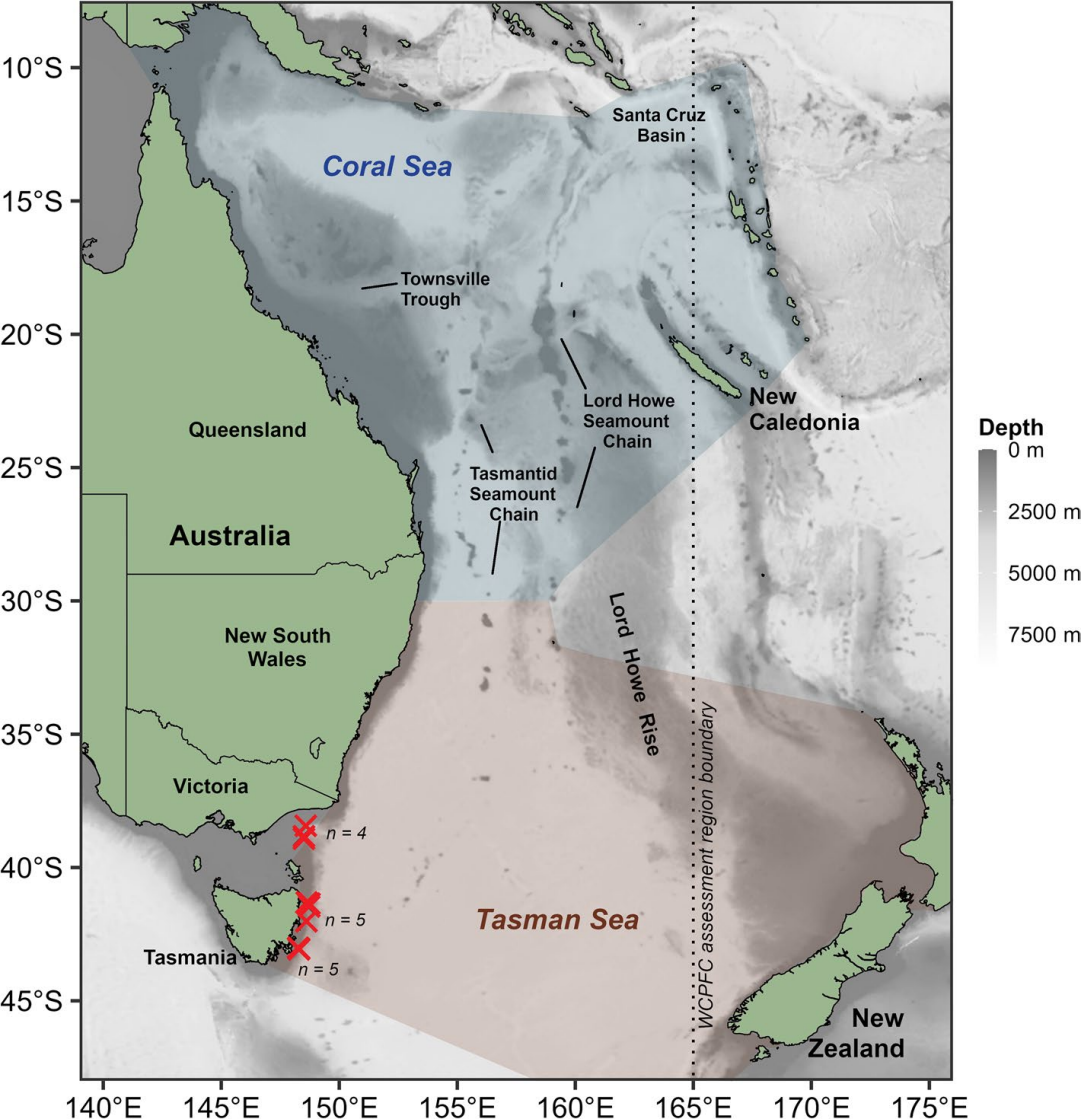
Southwest Pacific Ocean study area. Red ‘**×**’ markers indicate the location of swordfish tagging events (*n* indicates number of individuals tagged in each cluster of overlapping markers). The dotted line indicates the 165°E parallel separating WCPFC stock assessment regions. The red and blue tinted areas indicate the IHO boundaries of the Tasman Sea and Coral Sea^43^, respectively, and select bathymetric features are labelled according to^44^. Generated in R 4.2.1^67^ with ETOPO1 bathymetry^46^.

The degree to which longitudinal (east ↔ west) movements of swordfish provide connectivity across and between regions of the Pacific Ocean is a key research gap. To date, conventionally and electronically tagged swordfish have largely maintained a regional association to release sites and tagging studies to date have not recorded trans-Pacific crossings in temperate regions^8,12–16^. By contrast, genetic evidence is consistent with population connectivity across the temperate regions of the Pacific, which presumably would require some degree of longitudinal dispersal or migration of adult swordfish to maintain. For example, despite great distances separating sampled areas, no significant genetic differences have been found between swordfish sampled across temperate regions of the Pacific, nor between temperate regions of the northern and southern hemispheres^5^. Similarly, no evidence of differentiation was found in a SNP-based analysis of swordfish sampled across the southwest Pacific^17^. However, the most comprehensive tagging study of southwest Pacific swordfish to date found limited evidence of connectivity across the region^8^. Of 30 swordfish tagged with pop-up satellite archival transmitters (PSATs) in the vicinity of Australia (154°E–161°E) in^8^, only three were reported to move east of 165°E (dotted line; Fig. 1) and one east of 170°E into the vicinity of New Zealand. However, PSAT deployments in^8^ were concentrated off subtropical southern Queensland where commercial fishing effort is focused, so it is possible greater longitudinal movements occur among subsets of swordfish occurring elsewhere in the region. Resolving the degree of longitudinal movements of swordfish (particularly between the Tasman/Coral Sea and South Pacific region to the east) was recognized in the most recent stock assessment as a key research need to reduce uncertainty in the stock status^18^.

**Figure 2:**
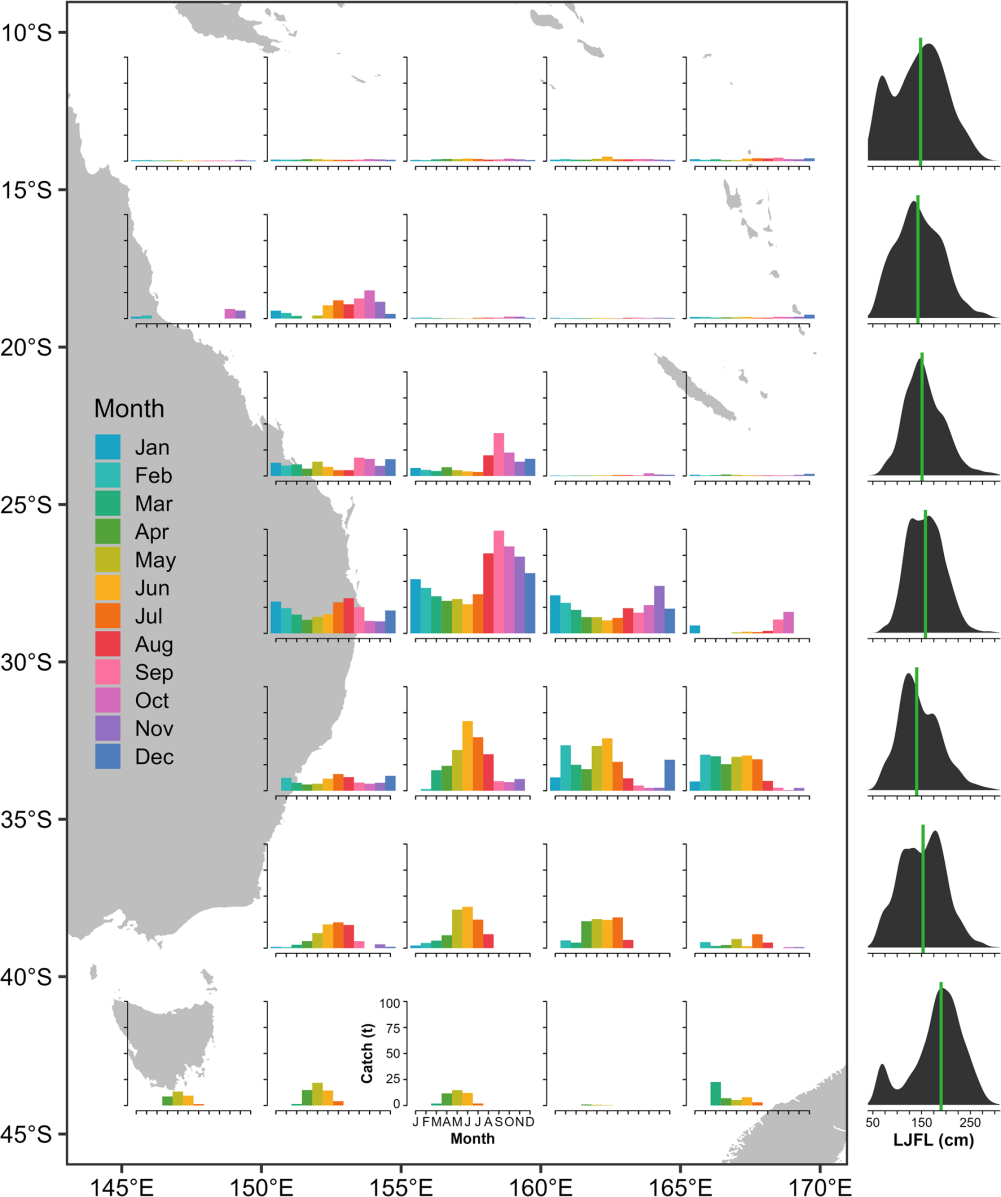
Mean annual commercial swordfish longline catch by month in 5° resolution grid cells, from 1980 to 2019. Right: Distribution and median (horizontal line) of lower jaw fork length data from 2010 to 2019, by latitude. Sourced from WCPFC public domain data (note that data contributed by less than three vessels per cell-month are redacted). Generated with R 4.2.1^67^.

Genetic and tagging evidence suggest some degree of latitudinal (north ↔ south) connectivity between the tropical and temperate areas within the southwest Pacific region (i.e., the Coral and Tasman Seas; Fig. 1), but a lack of connectivity between the southwest Pacific and the western and central Pacific Ocean to the north. Of the aforementioned 30 swordfish that were tagged in^8^ in the vicinity of Australia (between ~ 24°S–30°S), latitudinal displacements were largely between 20°S and 35°S, with a single swordfish crossing 40°S into cooler temperate waters. In contrast, in other regions where electronic tagging studies have been conducted, migration towards temperate or cold water to feed during summer months has been reported^12,19^. This migration appears more common among larger individuals, particularly females (which grow substantially larger than males), which are thought to then return to warmer waters to spawn. While tagging to date has found limited evidence of movement from the Coral Sea to cooler temperate waters of the southern Tasman Sea, available commercial catch data demonstrates swordfish have at least occasionally occurred in the Tasman Sea over the past 40 years, and that those caught at high latitude tend to be relatively large (Fig. 2). Further, the recent emergence of a recreational daytime swordfish fishery in southeast Australia demonstrates notably large swordfish occur seasonally in temperate areas of the southwest Pacific^20^.

In 2014, a small temperate Australian recreational fishery developed along the coasts of Victoria and Tasmania (38°S–43°S) due to the rapid adoption of the ‘deep-dropping’ fishing technique (i.e., targeting swordfish during the day with baits set well into the mesopelagic layer 300–700 m), and garnered international attention after yielding several swordfish line-class weight records within its first few years^20^. The unprecedented access to large, high-latitude swordfish provided by this fishery delivered an opportunity to investigate possible spawning-related migration and resolve the degree of longitudinal and latitudinal movement among this subset of the southwest Pacific swordfish population.

Some evidence suggests that the movements of swordfish are interspersed with periods of local association or site fidelity to particular areas^21,22^, perhaps linked to philopatry^23^. The behavior of fish returning to discernible bathymetric features (e.g., continental slope anomalies or seamounts) could increase susceptibility of the species to localized depletion. For example, as the Australian domestic longline fishery rapidly expanded in the late 1990s from southeast Queensland, a pattern of serial depletion was identified, where favorable catch rates for swordfish could only be maintained in new areas on the periphery of the expanding fishery and declined the longer an area had been fished^9^. The sequential decline trend was particularly evident in fishing areas that were associated with seamounts, but conversely, this trend was not noted for other target species of the fishery including striped marlin (*Kajikia audax*) and bigeye tuna (*Thunnus obesus*)^9^. This finding suggests that relative to that of other highly migratory species, the movement behavior of swordfish may result in slower replenishment from other regions. While the impact of the southeast Australian recreational fishery on the southwest Pacific swordfish population is presumably limited due to its present niche nature, the potential for susceptibility of swordfish to localized depletion is pertinent to the state-level management of the recreational fishery. Thus, quantifying movement behavior of swordfish in the region would inform recreational fishery assessment and management for what is typically a commercial target species managed at the Commonwealth (national) level.

Along with horizontal movements, the vertical movement patterns of swordfish are important considerations for management because they determine the degree of overlap with the distributions of fishing gear and potential bycatch species. Swordfish typically undergo a diel vertical migration from the upper-mid epipelagic zone at night (< 100 m depth), where most targeted catch of swordfish occurs, to deeper and usually well into the mesopelagic zone during the day (i.e., 300–800 m depth; 8, 13, 14, 24), and thus out of reach of most fishing gears. However, the vertical movements of swordfish during both day and night vary with environmental conditions. Swordfish are thought to use vertical movement to maintain a preferred amount of ambient light^15,25^, and greater water column light attenuation is linked to shallower daytime depth selection of tagged swordfish^14^. During the day, low dissolved oxygen or temperature may limit the depth or duration of dives into the mesopelagic zone^15,25^. As opportunistic predators, swordfish in some regions exhibit variable, shallower daytime behavior, likely linked to prey availability through the water column (e.g., mean daytime depth 234 m;^26^). Occasionally, swordfish exhibit daytime ascents to near the surface, or ‘basking’^14,15^, likely to recover body temperature^27,28^. Basking makes swordfish available for varying daytime periods^26,29^, facilitating regional harpoon fisheries^30,31^.

At night, moon phase has been shown to influence vertical distribution of swordfish, with tagged fish significantly deeper during bright full moons than dark new moons^24,32^. The shift in depth, in turn, appears to influence interactions with fisheries. Perhaps due to gear differences (e.g., depth of hooks or nets), the reported effects of moon phase on swordfish catch per unit effort range from peaking at the full moon^33^, new moon^34^, or other phases^35–37^. A better understanding of the environmental drivers of vertical movements of swordfish in the southwest Pacific will increase capacity to understand and manage fisheries dynamics by providing the capacity to capitalize on habitat segregation^26^, since swordfish are often caught with an assemblage of species of management and conservation concern^38,39^, the composition of which varies with depth of fishing gear^40–42^. Further, it is likely that environmental drivers like lunar phase can affect not just average night/day depth distributions, but differentially drive changes to the depth and timing of dives and ascents across the diel migration cycle. Despite availability of high-frequency depth data from archival tags, studies of swordfish movement to date have largely not examined effects of individual drivers on swordfish vertical movement behavior at fine temporal scale.

Here, we characterize the movement behavior of swordfish caught in the temperate southeast Australian recreational fishery with pop-up satellite archival transmitters to address several key knowledge gaps. First, we determine the extent of longitudinal and latitudinal movements of swordfish to assess evidence for connectivity both within the southwest Pacific region and between adjacent regions. Next, we model seasonal movement behavior to investigate evidence of spawning-related migration as well as evidence of regional residency and seasonal site fidelity. Finally, we characterize the vertical habitat use of swordfish and identify factors that shape the species’ vertical migration behavior across the diel cycle, and environmental drivers of day and night depth distributions.

## Methods

Swordfish were caught and released on the southeast Australian continental shelf break between the months of March and July, from 2014 to 2021. Fishing was focused on areas frequented by recreational fishers targeting swordfish, specifically east of Eaglehawk Neck, Bicheno, and St. Helens in Tasmania; and south of Lakes Entrance and Mallacoota in Victoria (Fig. 1).

### Swordfish capture, tagging, and release

All fish tagged in this study were captured using the daytime deep-dropping method, with baits being set on the seafloor at 350–650 m depth before being allowed to slowly drift up through the water column (see^20^ for details of fishing methodology). As swordfish were brought to the side of the vessel, they were held in the water and their condition was assessed with a modified ACESS scale to identify candidates suitable for release^20^. For each swordfish released, a pop-up satellite archival transmitter (MiniPAT; Wildlife Computers, Redmond, WA, USA) was attached. The tags were rigged with a Domeier nylon umbrella dart tag anchor^45^. The anchor was connected to the tag via a 200 kg breaking strain stainless steel multi-strand wire tether, covered in plastic heat-shrink and crimped to the corrodible release pin of the PSAT tag. Each PSAT was affixed in the musculature just below the dorsal fin using a purpose-made tagging pole, with the aim of inserting the anchor between two pterygiophores. After the tag was affixed, the weight of the fish was estimated and fish were then held alongside the vessel, which was moving forward at approximately 1–2 knots to provide gill perfusion until the fish freely kicked from the grip of the handler (“release”), and release location coordinates were recorded.

Each tag was deployed in ‘standby’ mode and programmed to activate when wet and at a depth of greater than 2.5 m. The tags recorded pressure (converted to depth in meters of seawater, m), temperature (°C), and light level (lumens). Tags deployed from 2015 onwards were programmed to detach from the fish after 250 days with a sampling interval of 10 min, with one fish programmed to detach after 365 days. The light attenuation coefficient was set at a constant 0.25. Alternatively, if the tag sinks to a depth greater than 1800 m or the depth of the tag does not change by greater than ± 2.5 m over a 2-day period, the tag would also detach from the tether. Once the tags detached from the fish, they floated to the sea surface where data was transmitted to the Advanced Research and Global Observation Satellite (ARGOS) system.

### Geolocation estimation

Geographic position estimates of tagged swordfish were derived at twelve-hour intervals using a state-space model accessed through Wildlife Computers proprietary software, Global Position Estimator 3 (GPE3). GPE3 uses the timing of dawn and dusk (as identified by the temporal profile of luminosity intensity recorded onboard the tag); and comparisons of the depth recorded on the tag versus bathymetric data at the estimated position^46^ and in situ sea surface temperature (SST; estimated when the tag was near the surface) versus remotely sensed reference SST data^47^. These data inform a diffusion-based movement model to generate time-discrete 0.25° resolution gridded probability surfaces. GPE3 includes a speed parameter (the standard deviation of modelled diffusion rate) to account for biological plausibility of animal movement speed when estimating sequential positions. To allow for additive effects of currents in the southwest Pacific (e.g., up to 1.5–2 ms^−1^ in the EAC and associated eddies^48,49^) on swordfish movement rates, the parameter was set to 3 ms^−1^.

### Behavioral state modelling

To investigate the dynamics of swordfish horizontal movement behavior, we estimated behavioral states and state-switching dynamics over time with a Hidden Markov Model (HMM) implemented largely with the R package *HMMoce*^50^. This approach is similar to GPE3 in estimating positions on a discrete grid based on PSAT data, but additionally estimates a joint probability of both location and behavioral state at each time step^51^. A state switching model with two states, a high diffusion state consistent with transitory or migratory behavior and a low diffusion state, consistent with restricted movement or residency, was fit as follows. We started with the 0.25° resolution twelve-hour GPE3 position likelihood grids for each tag, as these already account for in situ bathymetry-, light- and SST-based likelihoods. Behavior state parameters (σ_1_ and σ_2_, diffusion kernel standard deviation for each state; *p*_11_ and *p*_22_, probability of remaining in a state in the subsequent time step) were estimated with a genetic algorithm (noted by *HMMoce* authors to produce better parameter estimates than gradient-based optimization alternatives^50^) with R package *GA*^52^ by maximizing model log likelihood in an HMM filtering process^50,53^. The genetic algorithm was run with a population size of 200, probability of mutation of 0.12, and 120 maximum iterations, and model parameter optimization was constrained to 0.1–6.0 grid cells (0.25 × 0.25°) for σ_1_ and 0.001–1 grid cells for σ_2_, while state non-switching probabilities *p*_11_ and *p*_22_ were constrained between 0.02–0.98. The lower bounds of the *p* parameters were increased to 0.12 in the case of poor optimization results, which occurred in one run. After parameter estimation, HMM filtering and smoothing were carried out with *HMMoce* to yield posterior distribution estimates of location and behavior of each tagged swordfish at twelve-hour intervals.

### Vertical habitat use and movement behavior

Depth and temperature profile time series were visually inspected for anomalous data at the beginning and end of PSAT deployments (i.e., for post-release behavioral disruption or evidence of predation and consumption of the PSAT by a predator) and if present, data series were truncated accordingly. To characterize vertical movement behavior and habitat use of swordfish, a series of general additive models (GAMs) were constructed with the R package *mgcv*^54^. First, to characterize dynamic diel patterns in swordfish diving behavior, a GAM relating depth to smooth functions of covariates was fit to PSAT depth data (which is recorded at ten-minute frequency). As tag depth (in meters of seawater) is constrained to positive values, and variance is expected to increase with mean depth, the following model (herein ‘vertical behavior model’) was fit with a gamma error distribution and a log link function:

$${depth}_{i}=f\left({time}_{i}\right) + f\left({day}_{i}\right) + f\left({lat}_{i}\right) + f\left({moon}_{i}\right)+f\left({time}_{i},{day}_{i}\right) + f\left({time}_{i},{lat}_{i}\right)+f\left({time}_{i},{moon}_{i}\right) +f\left({time}_{i},{lat}_{i},{day}_{i}\right) +{ f}_{{Fish}_{i}}\left({time}_{i}\right) +{ f}_{{Fish}_{i}}\left({day}_{i}\right) + \zeta Fish + {\varepsilon }_{i}$$

where *time* is time of day in decimal hours UTC (i.e., 10–13 h offset from local time in the southwest Pacific such that 24-h periods begin close to noon); *day* is Julian day of year (rotated, such that 1 = March 1 and 366 = February 29); *l* is latitude (in decimal degrees), *moon* is lunar phase (in radians, such that 0 and 2π = new moon, and π = full moon, derived for each datum with the package *lunar*; Lazaridis 2022); ζ*Fish* is the random intercept for individual swordfish and ε_*i*_ is the residual error term. The type of smoothing functions (indicated by *f* ) used varied according to the nature of the covariates. The interactions of time of day with other covariates, modelled as tensor interaction smooth functions, were of primary interest but the interacting covariates were also included as main effects. The cyclical predictors (*time, day, moon*) were smoothed with cyclic cubic splines^56^, such that means and first derivatives of these smooths were ‘wrapped’ (e.g., so time of day was continuous from 24 to 0 h), and latitude was smoothed with a thin plate spline^57^. Individual-level smooth functions (*f*_*Fish*_) were included to account for any consistent individual differences in diel diving behavior or long-term vertical habitat use. To account for autocorrelation of sequential tag depth data, a first-order autocorrelation model was fit to the residuals of each tag, with the ρ coefficient chosen through model residual autocorrelation inspection with R package *itsadug*^58^. Smooths of time of day were allowed a basis dimension (*k*) of 48 to accommodate acute shifts in depth across time of day, which induced a high degree of model complexity since several interaction terms include *time*. Due to high model complexity, to ensure computational feasibility the model was fit with the *mgcv* function bam(), with fast restricted maximum likelihood and discretization of covariate values^59^. To assess whether the model was over-fitting the data, it was refit with 10% of the data withheld as a validation set. The retrained model was then used to predict depth values of the validation set, to examine whether the total deviance it explained among the validation set withheld from training was notably worse than that explained among the training data.

To investigate environmental drivers of swordfish vertical habitat use, separate GAMs were built for day and night with median depth of each period as a response. Modelling daily median depths greatly reduced model complexity relative to the full vertical behavior model that included time of day interactions, and it also allowed modelling of independent effects of covariates on depth during daytime and night-time without the constraint of an autocorrelated relationship across each day. Daytime and night-time median depths were assigned the geographic coordinates of the most-likely (point) position estimated by GPE3 during each day or night period (positions were estimated at 00:00 and 12:00 UTC, corresponding to near mid- day/night in the study area respectively), and these coordinates were used for extraction of other covariates. Sun positions were estimated based on date and daily estimated location with the R package *suncalc*^60^. Based on evidence from the vertical behavior model and visual inspection of individual daily depth data series, the rapid shifts in depth consistent with vertical migration were observed to occur in the period spanning golden hour to astronomical twilight (or vice versa), and data recorded during this period (considered ‘twilight’) were omitted from calculating day and night median depths. Depth data were considered to have occurred during daytime if recorded between the end of morning golden hour and the start of evening golden hour (sun elevation > 6° above horizon), and during night-time if between the end and beginning of consecutive astronomical twilight periods (sun elevation > 18° below horizon).

Water temperature (°C), sea surface height (m), and mixed layer depth (m) were sourced from BRAN2020, an ocean reanalysis model that assimilates observation data to provide gridded estimates of data (daily 0.1° resolution) throughout the water column^61^. Temperature from the top (2.5 m) depth layer (i.e., SST) and the 545 m depth layer were included as covariates, as these are roughly consistent with the bounds of habitat exposure of swordfish during the night and day, respectively. The diffuse attenuation coefficient at 490 nm (K490, m^−1^) was sourced from Level 3 MODIS AQUA data at monthly, 4 km spatial resolution^62^ from ERDDAP^63^ with the R package *rerddapXtracto*^64,65^. K490 is a measure of how much light intensity is lost to turbidity in the water column, such that K490^–1^ is the distance in meters at which light is reduced log-fold (i.e. by ~ 73%). Due to high levels of missing values, rather than extracting K490 at most likely location point estimates, mean values were aggregated across the 50^th^ percentile contour of the GPE3 posterior likelihood grid of each 12-h timestep, weighted by position likelihood across the grid.

Hierarchical GAMs were built with both global and group-level smooth functions for each covariate to account for individual variation in responses^66^. As night-time depth is typically near the surface and thus bound to positive values, its logarithm was modelled as a response. Full median night and day depths models initially fit were:

$$\genfrac{}{}{0pt}{}{\mathrm{log}\left({nightdepth}_{i}\right)}{ {daydepth}_{i}}=f\left({moon}_{i}\right)+f\left({K490}_{i}\right)+f\left({temp}_{2.5i}\right)+f\left({temp}_{545i}\right)+f\left({mld}_{i}\right)+f\left({ssh}_{i}\right) +{f}_{{Fish}_{i}}\left({lat}_{i},{lon}_{i}\right)+\zeta \mathrm{Fish}+{\varepsilon }_{i}$$

Non-cyclical variables were smoothed with thin-plate splines and *moon* a cyclic spline as above. To account for individual variability without inducing artifact beyond the range of each individual’s spatial extent, a stationary spherical gaussian process with a range of 0.25° was used to model individual-level location terms of *lat* and *lon* (longitude)^56^. As this approach assumes the units of geographic covariates represent the same distance, *lon* was scaled by the cosine of median *lat* in the study area to approximate isotropy. Model selection was performed with the double penalty approach so that terms with negligible effect could be shrunk to zero^56^, and terms that were penalized to zero were removed during model fitting. After selection of global covariate terms, model selection was repeated with the inclusion of fish-level smooths to ensure relationships were robust after accounting for individual variability. Fish-level smooths were fit with a penalty order *m* = 1 to reduce concurvity with global terms^66^. The global terms that were shrunk from the model upon refitting were removed along with the corresponding fish-level terms.

All analyses (excluding GPE3 geoposition estimation) and production of figures was conducted in R version 4.2.1^67^.

All experimental protocols used in this study were approved by the University of Tasmania’s Animal Ethics Committee (project numbers A0014679 and A0017003). All methods were carried out in accordance with relevant guidelines and regulations. The methods and results are reported in accordance with ARRIVE guidelines (https://arriveguidelines.org/).

## Results

A total of 14 PSAT tags were deployed on swordfish during the study (Fig. 1). Two fish died soon after release and one tag did not report^20^. The remaining 11 tags were deployed for durations between 56 and 250 days (Table 1). Three tags reached full programmed deployment durations and eight tags reported prematurely, including three due to PSAT release pin failure, and one (SC0007) which reported 185 days after release but recorded consistent, elevated temperature and irregular depth data during the final nine days, consistent with being consumed by a heterothermic predator (see Supplementary Fig. S1). In total, PSAT data were recovered on 1861 unique swordfish-days.

**Table 1:** Deployment and transmission information from pop-up satellite archival tags deployed on Swordfish caught adjacent to southeast Australia caught using recreational fishing methods. Fish IDs in italics are not included due to post-release mortality or because the PSAT did not report (DNR).

Fish ID	Est. mass (kg)	Deployment				Pop-up transmission			
		Date	Latitude	Longitude	Program duration (days)	Date	Latitude	Longitude	Actual duration (days)
SC0001*	180	21/06/14	43° 01′S	148° 15′ E	180	23/06/14	43°10′ S	148°15′ E	2
SC0004	100	10/03/16	43° 03′ S	148° 16′ E	250	15/11/16	18°54′ S	152°15′ E	250
SC0007†	280	08/04/16	41° 20′ S	148° 37′ E	250	10/10/16	29°44′ S	154.08′ E	185
SC0008	140	12/04/16	41° 19′ S	148° 40′ E	250	15/10/16	25°29′ S	173°42′ E	186
SC0010*	270	12/04/16	43° 19′ S	148° 39′ E	250	18/04/16	41°54′ S	148°43′ E	6
SC0012	80	31/05/16	43° 01′ S	148° 17′ E	250	05/02/16	43°58′ S	149°04′ E	250
SC0013	50	31/05/16	43° 01′ S	148° 17′ E	250	11/11/16	40°50′ S	156°33′ E	164
SC0014	115	02/06/16	41° 17′ S	148° 40′ E	250	07/02/17	36°26′ S	152°36′ E	250
SC0016	350	05/04/17	43° 03′ S	148° 16′ E	250	10/08/17	38°48′ S	148°24′ E	127
SC0019	90	18/05/19	38° 50′ S	148° 28′ E	250	13/07/19	38°15′ S	149°22′ E	56
*SC0021*	125	08/06/19	38° 54′ S	148° 31′ E	250	–	–	–	DNR
SC0023	160	01/06/21	38° 27′ S	148° 35′ E	250	05/09/21	38°54′ S	149°56′ E	96
SC0024	90	02/06/21	38° 27′ S	148° 35′ E	250	05/01/22	29°19′ S	156°01′ E	217
SC0025	90	08/07/21	38° 08′ S	149° 25′ E	365	26/09/21	35°56′ S	154°20′ E	80

*Post-release mortality, see^20^.

^†^Pop-up occurred 21/10 however data after 10/10 are consistent with predation.

### Horizontal movements and behavior

**Figure 3:**
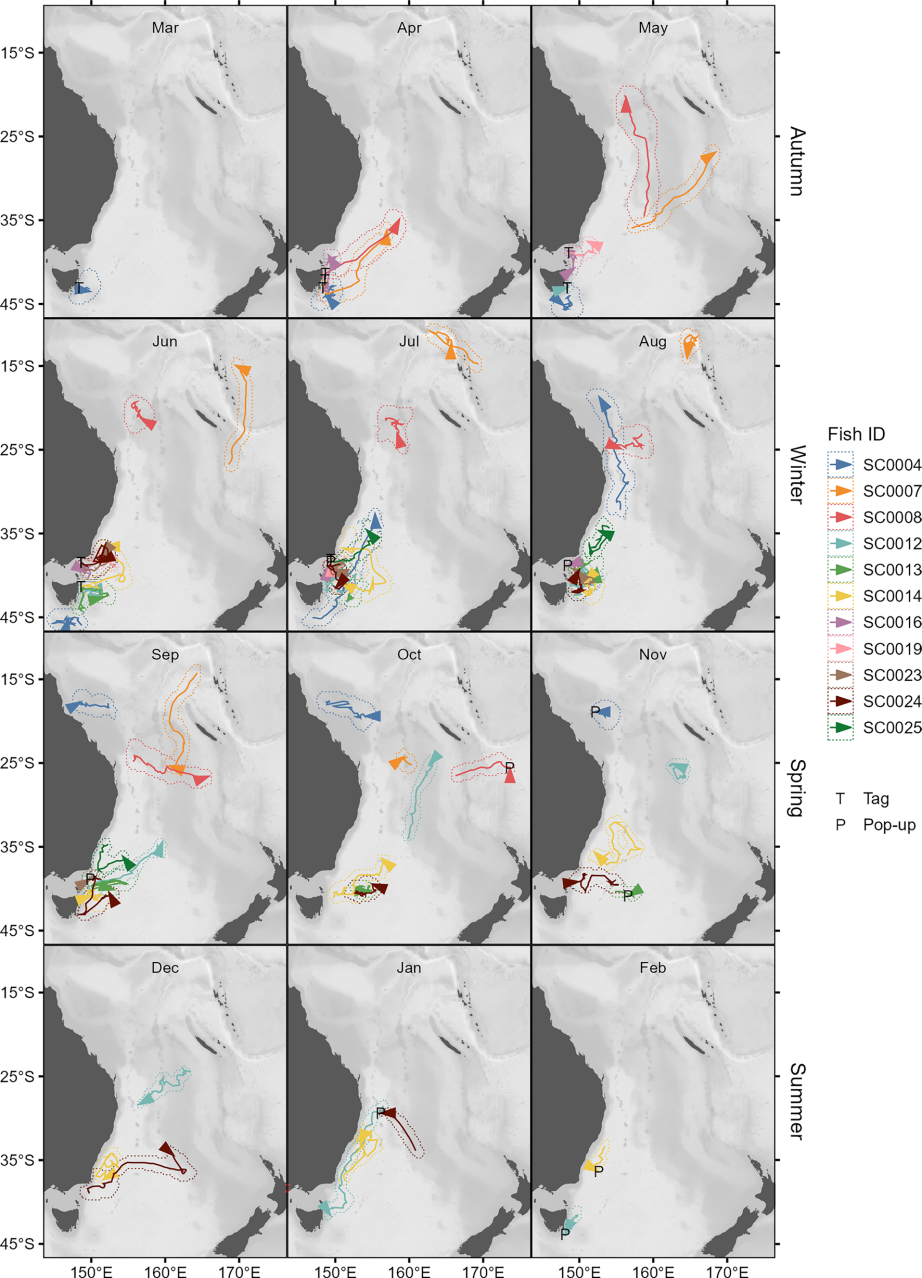
Monthly movement trajectories of eleven swordfish. Trajectories are inferred from most-likely geolocations estimated from pop-up satellite archival transmitter (PSAT) data by the GPE3 algorithm. Arrowheads indicate the position of each fish at the end of a month (or at tag pop-up, if before the end of a month). Dotted lines around each trajectory indicate the 95th percentile contour of aggregated 12-h posterior likelihood grids output by GPE3. T and P indicate the location of tagging and pop-up (the first ARGOS transmission after tag release). Note that tags were deployed across multiple years (2016–2021). Generated in R 4.2.1^67^ with ETOPO1 bathymetry^46^.

Geolocation estimates revealed tagged swordfish dispersed as far north as 10°56′S and as far east as 173°39′E during PSAT deployments (Fig. 3), with maximum displacement from tagging location during deployments ranging from 202 to 3275 km (median: 1164 km).

**Figure 4:**
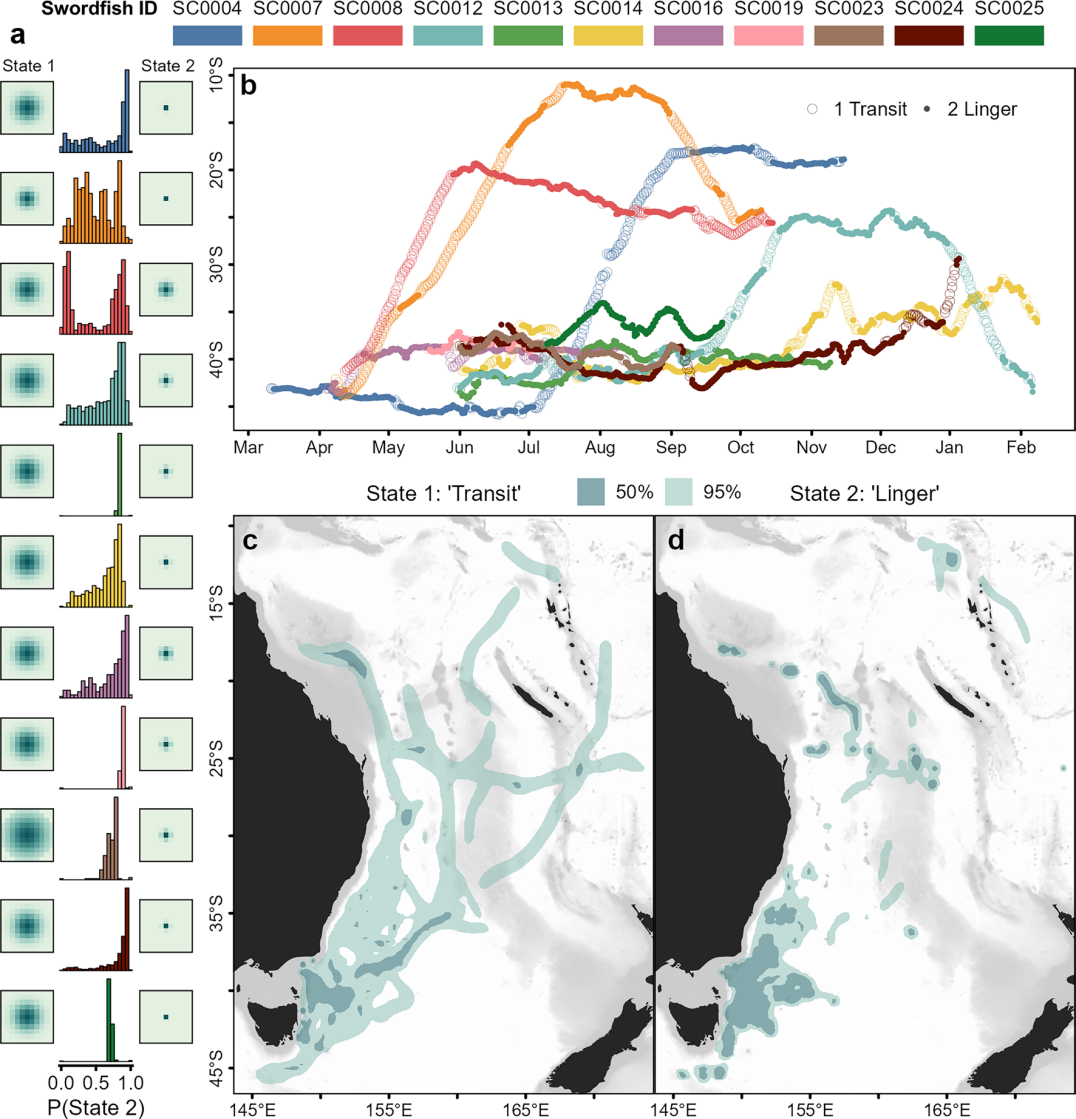
Swordfish movement behavior state Hidden Markov Model results. (**a**) The diffusion kernels fit for State 1 (‘transit’; *left*) and State 2 (‘lingering’; *right*) for each fish, with histograms of the probability of State 2 assignment at each 12-h time step. For reference, the extent of State diffusion kernel grid images is 2.75°. (**b)** Most-likely latitudes of each swordfish by day of year at 12-h intervals, by estimated behavior state. Open circles indicate estimated transit behavior at a given time point (i.e., probability of assignment to State 2 < 0.5), while closed circles represent lingering behavior (prob. State 2 assignment ≥ 0.5). Note that PSAT deployments across multiple years are depicted. Bottom: Expected residency distributions for behavior States 1, transit (**c**) and 2, lingering (**d**). Darker shaded area represents core distribution (50th percentile) contour and lighter shaded area 95th percentile contour.

Behavior switching model parameters were optimized with consistent diffusion parameters (σ) across individuals for each of the two behavior states of σ_1_ = 1.69 ± 0.10 (mean ± se) and σ_2_ = 0.49 ± 0.06 (Fig. 4a, Supplementary Table S1). Probabilities of state persistence were optimized at P_11_ = 0.62 ± 0.09 and P_22_ = 0.87 ± 0.02, consistent with sustained periods of limited regional-scale movement (State 2, herein ‘lingering’), interspersed among some fish with periods of transitory, often cross-region movement (State 1, herein ‘transit’).

**Figure 5:**
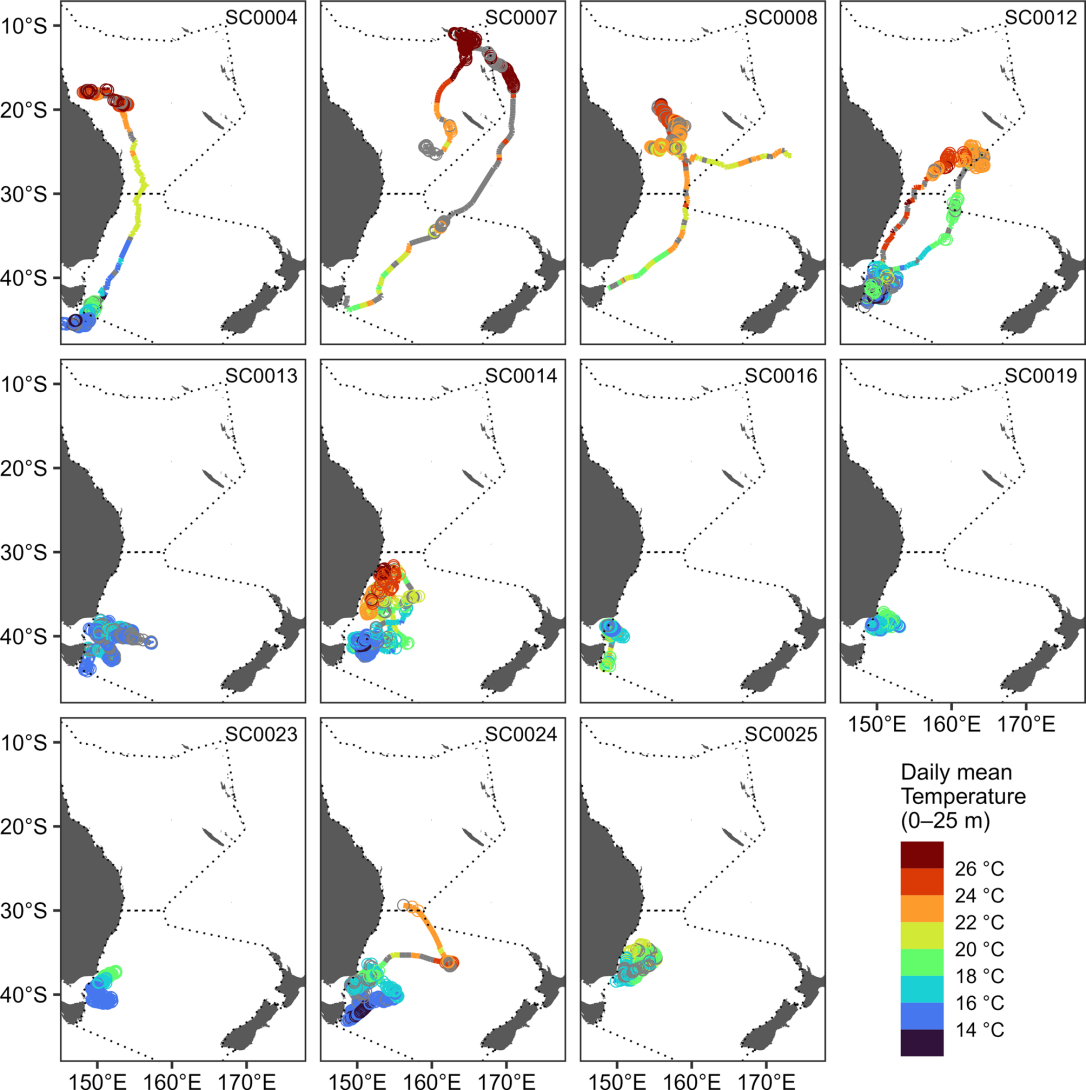
Average daily near-surface (0–25 m) water temperatures recorded by PSAT tags deployed on swordfish, by 12-h most-likely geolocations. Grey indicates day periods with missing temperature data. Circles indicate 12-h periods where the horizontal behavior model estimated the individual was lingering (state 2). Dotted lines indicate the boundaries of the Coral and Tasman Seas (top and bottom respectively). A shift from equatorward transit to lingering behavior in the Coral Sea was observed once near-surface water temperatures of 23–27 °C were encountered (i.e., potential spawning habitat). Generated in R 4.2.1^67^.

Broadly, two patterns of seasonal horizontal movement are discernible, distinguished by the presence of protracted equatorward migration. Five swordfish (SC0004, SC0007, SC0008, SC0012, and SC0024) left the temperate waters off southeast Australia in which they were tagged and transited into the Coral Sea, with the former four reaching minimum latitudes of ~ 11–24°S (Figs. 3, 4b). Timing of equatorward migration varied: two fish transited to lower latitude in April, just after tag deployment, while the other two transited after approximately five months of lingering at high latitudes, in July and September. However, all four individuals demonstrated lingering behavior for several months after transiting to the Coral Sea, and sea surface water temperatures at the initiation of lingering were 23–27 °C (Fig. 5). The single fish (SC0012) still carrying a PSAT after November transited back to temperate latitudes by the following January and resumed lingering behavior before PSAT release within 120 km of the location it was tagged, 250 days prior (Fig. 4b, Table 1), potentially indicating seasonal migration between the Tasman Sea and Coral Sea.

The other broad pattern consisted of predominantly or solely lingering behavior at high latitude, and was demonstrated by seven fish, none having moved into the Coral Sea (north of 30°S) for the duration of their PSAT deployments (Fig. 3). Four of these fish (SC0013, SC0016, SC0019 and SC0023) remained in temperate waters south of 37°S, and another (SC0025) remained south of 34°S for the entirety of deployments of 56 to 164 days duration (Table 1). The remaining two fish (SC0014 and SC0024) were slightly less restricted in latitudinal movement, with some transitory behavior recurring around 30–40°S rather than protracted equatorial transit, apart from the final week prior to SC0024’s tag release in which the fish rapidly transited northwest (Figs. 2, 4).

**Figure 6:**
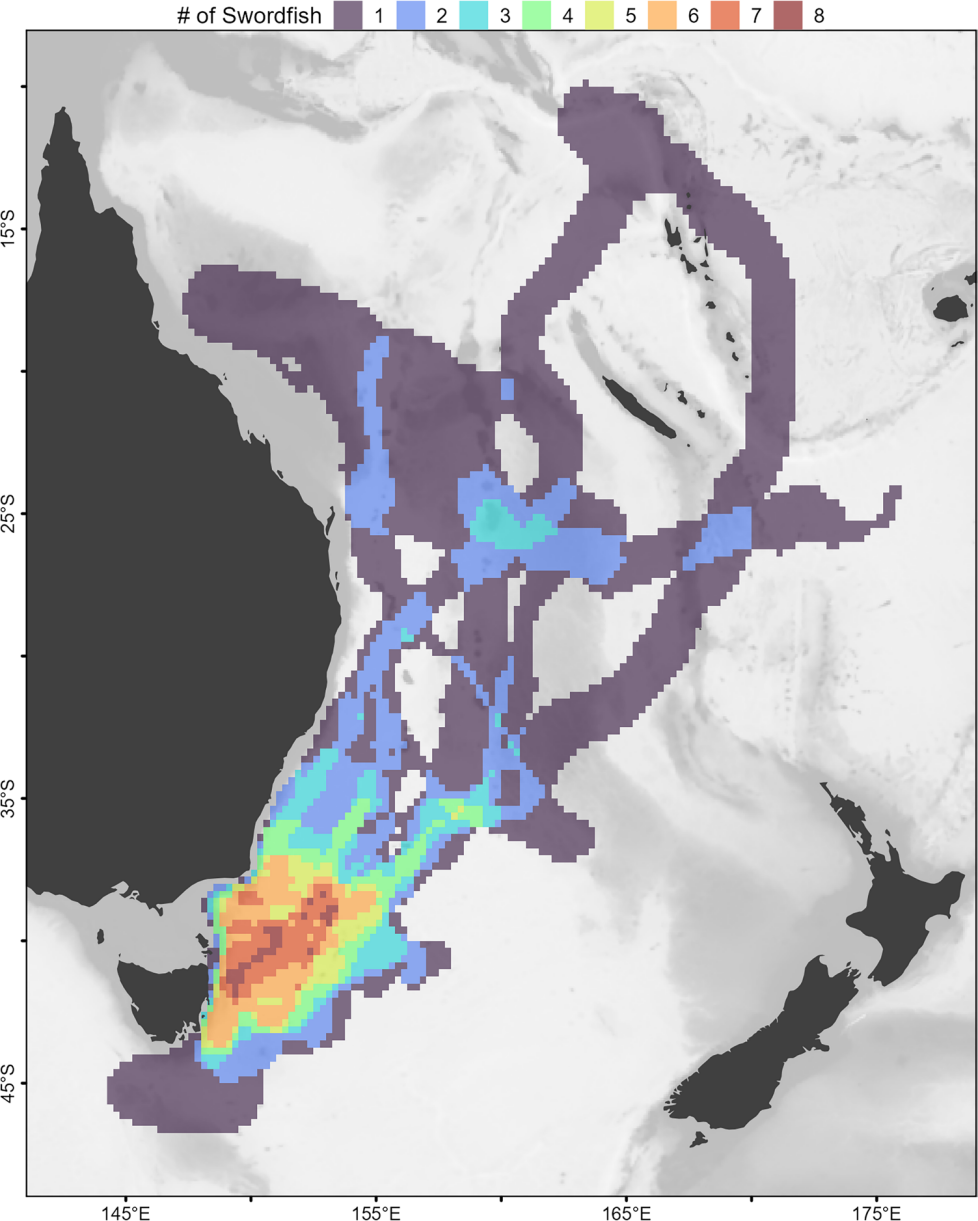
Spatial histogram of swordfish occurrence. Colors represent the number of individual swordfish with geolocation likelihoods (95th percentile contour of GPE3 likelihood grid across deployment) that occurred within a given at 0.25° grid cell. Generated in R 4.2.1^67^ with ETOPO1 bathymetry^46^.

The core residency distribution of transit behavior (Fig. 4c) reveals a discernible corridor heading northeast from the vicinity of the southeast Australian continental shelf break where tags were deployed. This corridor, which was used by four of the swordfish when transiting equatorward (Fig. 6), continues to the western edge of the Lord Howe Rise. At this point, transiting swordfish either continued north along the western edge of the Rise or continued northeast, traversing it. An additional, more diffuse corridor was discerned to the west, where four swordfish had transited at a north-northeast direction (Fig. 4c). Along with the section of the continental shelf in close proximity to PSAT deployment locations, the lingering (State 2) core residency distribution (Fig. 4d) included several discrete patches of habitat which were used by one to three tagged swordfish (Fig. 6) and several appear to be co-located with various bathymetric features. These include an area to the east of Tasmania in the proximity of a chain of seamounts (~ 153°E); to the north, directly east of Jervis Bay (35°S); farther north in the Coral Sea, in close proximity to the Tasmantid and Lord Howe Seamount Chains, the eastern edge of the Lord Howe Rise, the Townsville Trough, and the Santa Cruz Basin (see labels in Fig. 1).

### Vertical movement behavior and habitat use

**Figure 7:**
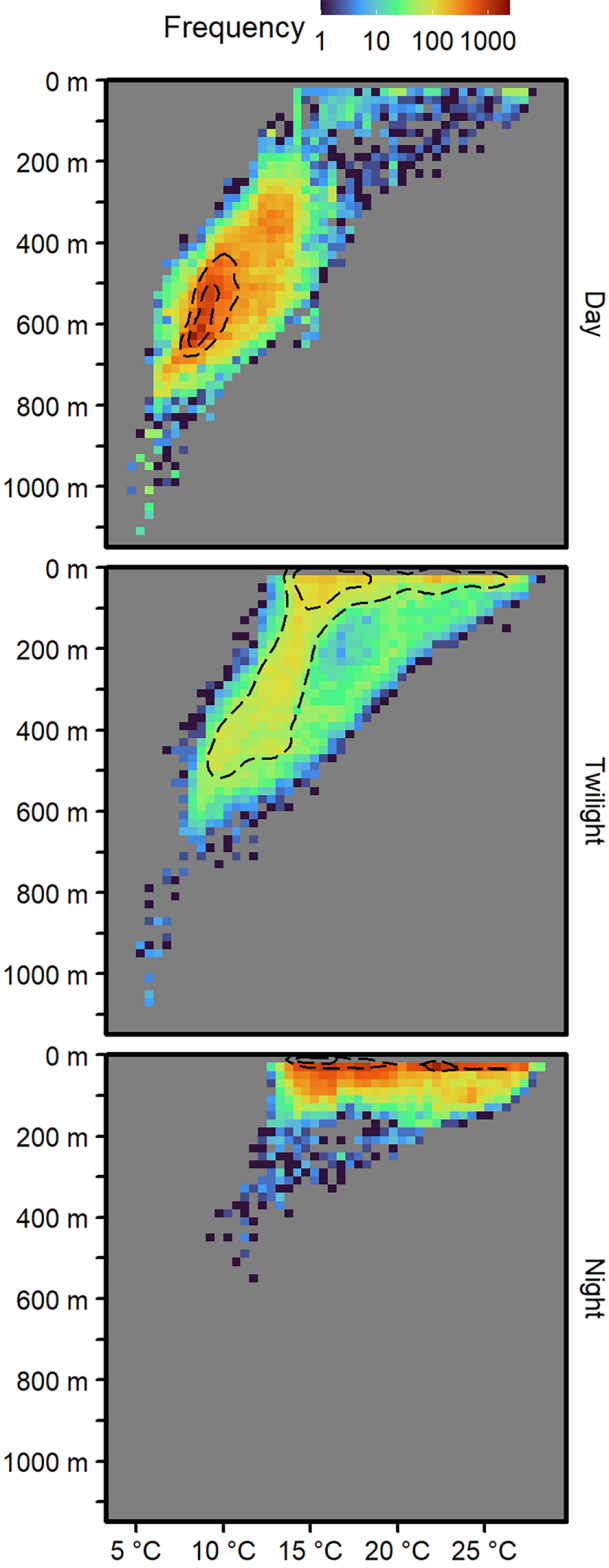
Histograms of co-recorded swordfish PSAT water temperature and depth data, during each of daytime, twilight, and night-time periods, where twilight spans astronomical twilight and golden hour periods (sun elevation between −18 and 6°). Dotted lines are density contours. Generated in R 4.2.1^67^.

Recovery of individual PSAT temperature and depth data series was 73% on average (range: 59–97%, see Supplementary Table S2), and was not significantly related to length of deployment (Supplementary Fig. S2). The first day of depth data post-tagging was removed for three fish and a longer period truncated for a fourth (SC0023), which exhibited approximately two weeks of abnormal diving behavior post-release (Supplementary Fig. S1). Minimum and maximum depths recorded during deployments ranged across individuals from 0–2 m and 680–1400 m (Fig. 7).

Swordfish predominantly exhibited normal diel vertical migration, with individuals descending to the mesopelagic zone (> 200 m depth) during the daytime on 99.5% of swordfish-days and ascending to the epipelagic zone (< 200 m depth) during the night-time on 100% of swordfish-days (Fig. 7). On 83% of swordfish-days, daytime depth data recorded was entirely in the mesopelagic zone, as was 96.7% of daytime depth data recovered across all days. There were some deviations from the normal diel vertical migration pattern of shallow at night and deep during the day, as daytime depths in the top 50 m of the water column were recorded on a median of 4.6% of swordfish days (ranging from 0–64.1% across individuals). However, depths of ≤ 3 m were only recorded on 0.19% of days (of the days on which daytime depths of < 50 m were recorded, 1.4%). PSAT depth rarely exceeded 1000 m, having only been recorded during single events for SC0004 and SC0007 and on three days for SC0014. Minimum and maximum temperatures logged ranged across individuals from 4.4–7.8 °C and 18.5–29.0 °C (Fig. 7).

**Figure 8:**
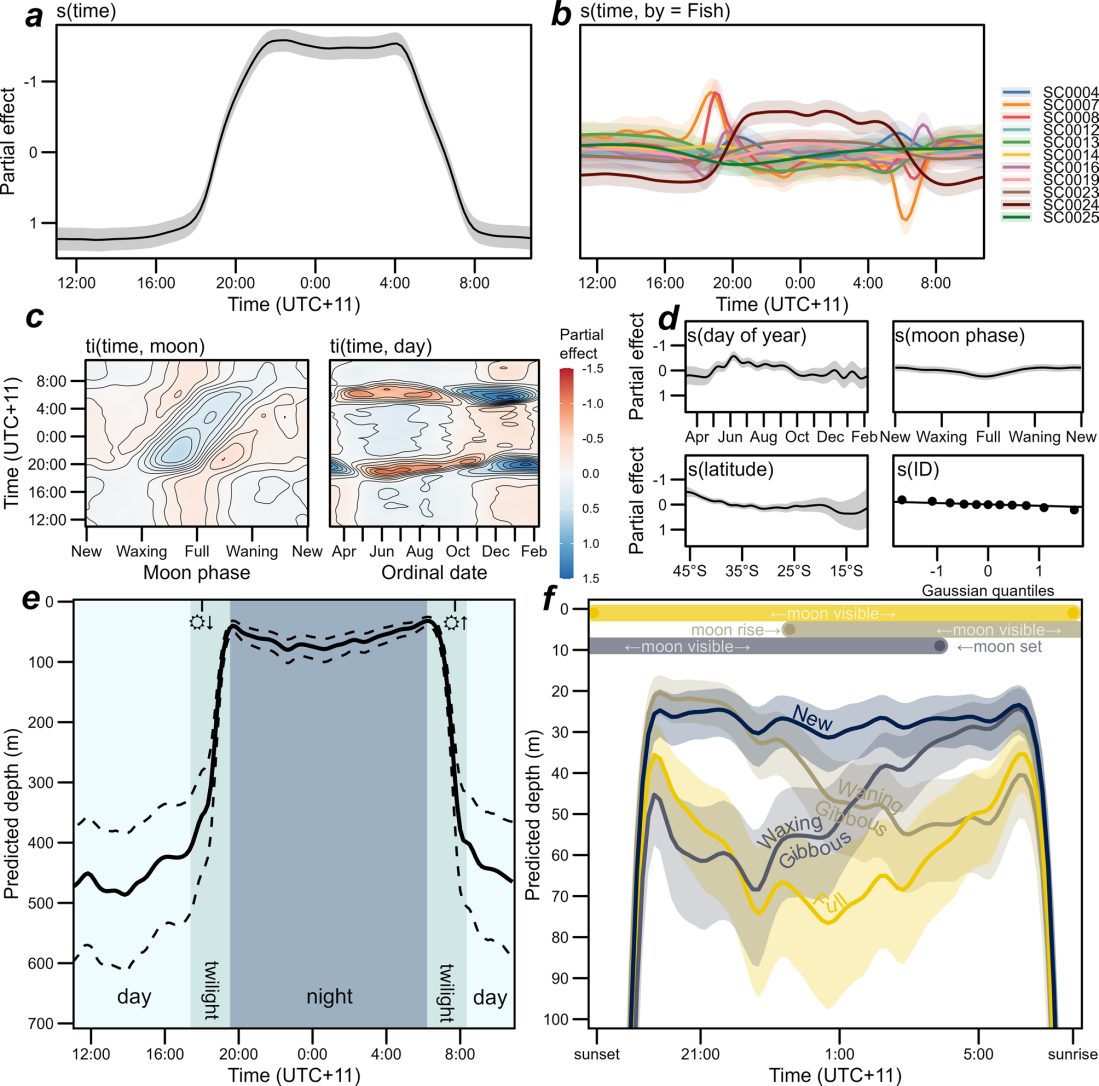
Swordfish vertical behavior general additive model results. Time of day is presented as local time in the study area (UTC + 11). Partial effects are on the link function (log) scale, positive effect values represent increasing depth. Time of day was by far the strongest predictor of depth (**a**), with the cubic cyclic smooth of time of day illustrating a consistent crepuscular shift in depth. The pattern was largely consistent across individual swordfish (**b**), although two migrated from depth at night consistently earlier and another was consistently shallower at night and deeper during the day (SC0024). The interaction functions of time of day and day of year, and time of day and moon phase (**c)** exhibit effects consistent with day length driving vertical migration timing and moon phase affecting depth at night. Smooth functions of main effects included (**d**) indicate limited evidence of consistent effects on depth independent of time of day, although depth across the diel cycle may decrease during winter and at high latitude. Bottom: Model predictions of depth across time of day overall (**e**), and at night, during new, full, and 3/4 full moon phases (**f**) with other covariates held constant at median covariate values (153.7°E, 38.6°S, Aug 13). The shaded regions of panel (**e**) indicate the periods encompassing twilight and golden hour (i.e., sun elevation −18° to 6°) and the tick marks at the top of the plot indicate sunset and sunrise time at the constant covariate time and latitude. Dotted lines (and shaded areas in panel (**f**) indicate 95% credible intervals. The bars at the top of panel **f** indicate the timing of moonrise and moonset during the ¾ full and full moon phases nearest median covariate values (see Supplementary Figs. S3 and S4 for predicted depths during half-moon phases and details on moon visibility across phase). Generated in R 4.2.1^67^.

Total deviance explained by the vertical behavior model was 0.836 (the model summary table can be found in Supplementary Table S3). Refitting on 90% of the data and predicting the withheld 10% validation set explained 0.835 and 0.832 respectively, suggesting the model does not overfit the data. Overall, the vertical behavior model revealed a consistent normal diel vertical migration (Fig. 8). The smooth function of time of day was by far the most important predictor of depth, consistently shallower in the water column during the night and greater during the day (Fig. 8a). The group-level effect of time of day varied from the global effect for some individuals, but with differences varying across time of day (Fig. 8b) rather than consistent shifts in depth (Fig. 8d). Notably, however, SC0007 and SC0008 consistently migrated both to and from depth consistently earlier in the morning and evening, respectively; SC0024 uniquely displayed a more pronounced vertical migration—deeper during the day and shallower at night (Fig. 8b). The interaction terms of time of day with day of the year and latitude are both consistent with an effect of the timing of sunrise and sunset on depth during the twilight periods of the day: swordfish are shallower for more hours of the day during the austral winter, and deeper for more hours during summer (Fig. 8c). However, there was little evidence of consistent effects of day of year, moon phase or latitude on depth independent of the time of the day, with the possible exception of being consistently shallower at high latitude (Fig. 8d) in the vicinity of the southernmost tagging locations (Fig. 1). Altogether, the model demonstrates swordfish undergo consistent crepuscular shifts in depth, well approximated by the twilight period spanning astronomical twilight and golden hour (sun elevation −18° to 6° relative to the horizon) during both matutinal descent into and vespertine ascent from the mesopelagic zone (Fig. 8e). Notably, there was a significant interaction on depth at time of day across lunar phase (Fig. 8c).

The vertical behavior model estimated a shallow consistent swordfish depth profile of 25–30 m at night during new moons, while during full moons, ascent to nearly as shallow (~ 40 m) were predicted at the beginning and end of night-time, but with depth increasing steadily until peaking in the middle of the night (70–75 m) before decreasing again until just before the rapid matutinal descent into the mesopelagic (Fig. 8f). Predicted depth profiles at intermediate lunar phases up to half-moon were not discernible from new moons (Supplementary Fig. S3), however at waxing gibbous phase (i.e., ¾ full), predicted depth across time of night was consistent or deeper than the full moon at the beginning of the night, then becoming shallower until consistent with the new moon at the end of the night, and the converse was predicted during the waning gibbous. These mirrored shifts in depth during gibbous moons coincide with the timing of moonrise and moonset on the respective waxing and waning phases (see Figs. 8f, Supplementary Fig. S4).

**Figure 9:**
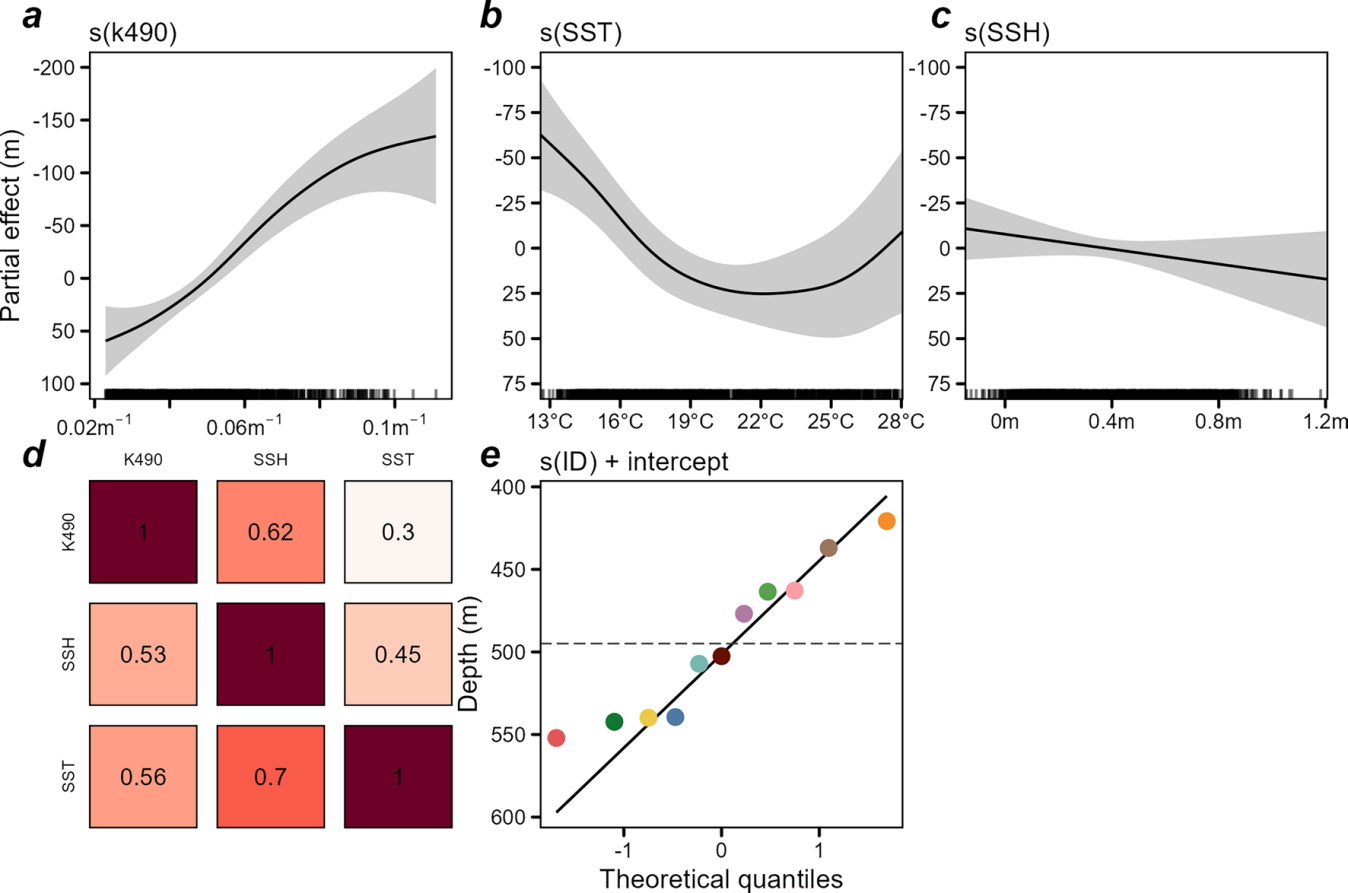
General additive model of drivers of swordfish daytime vertical habitat use. Top: partial effects in meters below surface (i.e., positive numbers indicate greater depth) for smoothed functions of light attenuation coefficient (**a**), sea surface temperature (**b**), and sea surface height (**c**). Panel (**d**): Pairwise observed concurvity of global model terms. Concurvity values (0–1) indicate the degree to which the smooth terms indicated by columns are dependent on (i.e., could be replaced by) terms indicated by rows. Panel (**e**): Individual swordfish random intercepts (global model intercept 494.9 m represented by the horizontal dashed line). Point colors correspond to individual fish per Figs. 3, 4, 8, 10. Generated in R 4.2.1^67^.

The median daily daytime depth model explained 0.650 of total deviance and yielded an overall estimate of median daytime swordfish depth of 494.9 m (460.4–529.5 m; Fig. 9; see model summary in Supplementary Table S4). There was no evidence of effects of moon phase, temperature at 545 m, or mixed layer depth on daytime depth, as these terms were penalized out of the model during fitting. The light attenuation coefficient K490 was the most important factor influencing daytime depth, with greater K490 (i.e., greater turbidity) resulting in shallower median swordfish depth by 195 m across the range of K490 values observed (0.02 to 0.11 m^−1^; Fig. 9d). The model also provided evidence of positive relationships between daytime depth with sea surface temperature (especially across 13–20 °C i.e., the temperate Tasman Sea) and to a lesser degree, sea surface height (Fig. 9a,b). The smooth of sea surface height was moderately concurve on sea surface temperature (Fig. 9e), likely due to the correlation between the oceanographic conditions with latitude. However, refitting with the sea surface height term removed did not notably change the influence of the other covariates (Supplementary Figure S5). There was a fair degree of fish-level variability across the fish-level intercepts, with individual swordfish varying from the global mean daytime depth by 74.2 m shallower to 57.1 m deeper (Fig. 9e).

**Figure 10:**
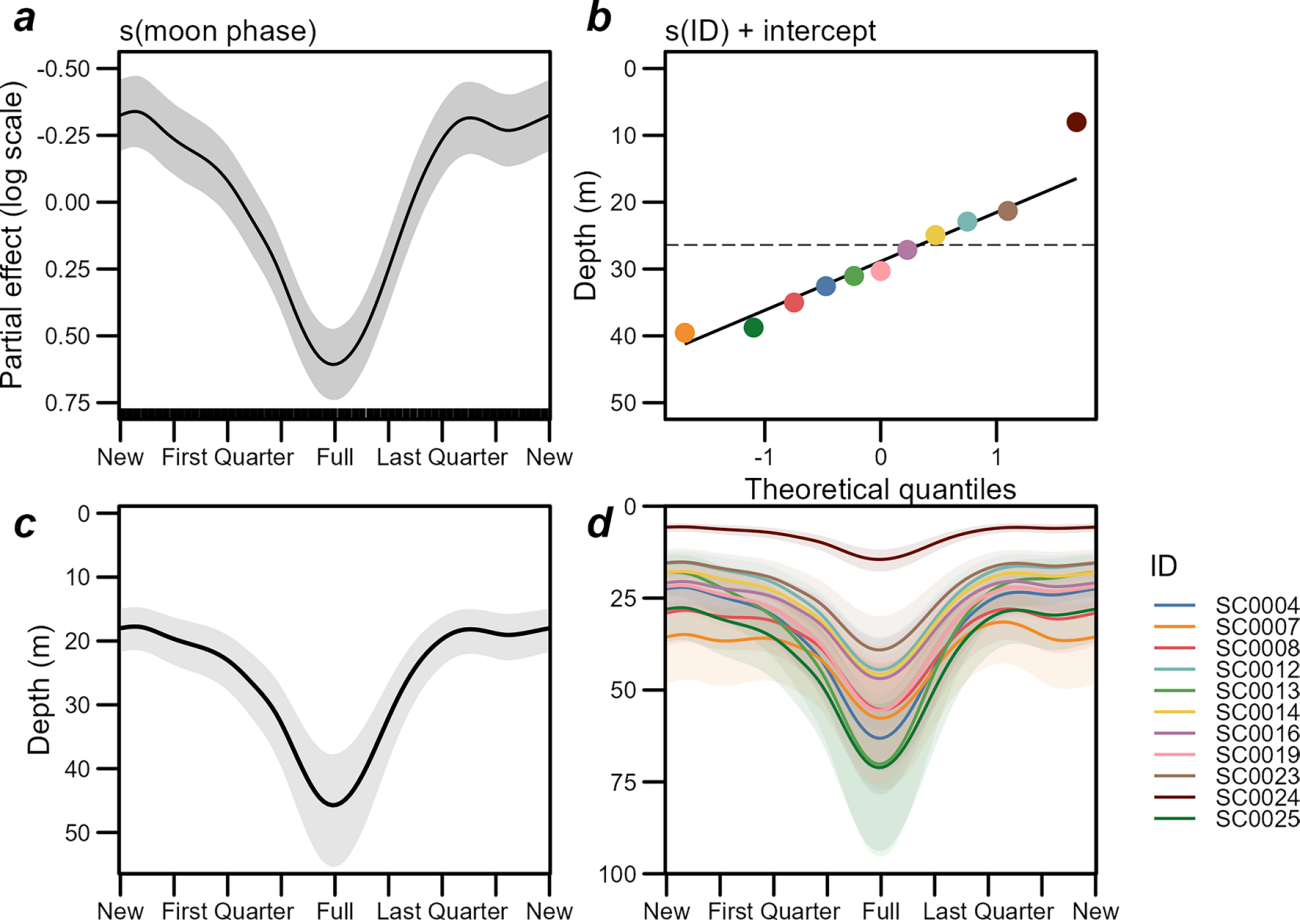
General additive model of drivers of median daily swordfish night-time log(depth). Top: partial effect on log scale of smooth functions of moon phase (**a**); and back-transformed random intercepts for individual fish (**b**). The dashed line indicates the model intercept (26.4 m, 95% CI 20.0–34.8 m). Model predictions by moon phase, overall (**c**) with random group-level smooths excluded, and at the level of each individual swordfish (**d**). Translucent bands indicate credible intervals. Generated in R 4.2.1^67^.

The night-time depth model yielded evidence of an effect of moon phase while all other environmental predictors were penalized out of the model, with total deviance explained of 0.465 (Fig. 10a; see model summary in Supplementary Table S5). The model estimated an overall estimate of median night-time swordfish depth of 26.4 m (20.0–34.8 m) while individual-level intercepts ranged between 8.0 m to 39.5 m (Fig. 10b). Nightly median depth was similar across moon phases from last quarter to first quarter (i.e., while between new and half full), but between first and last quarters there was a pronounced increase in median night-time depth that peaked at the full moon (Fig. 10c), further supporting the effect demonstrated by the diel diving behavior model (Fig. 8). While all individual swordfish were predicted to respond to moon phase, the magnitudes of the fish-level smooths of moon phase varied thus the predicted strength of response at the full moon did as well (Fig. 10d).

## Discussion

Swordfish in the Pacific Ocean are currently managed at a broad spatial scale, but the ecological reality of management boundaries and degree of connectivity within them remains uncertain. This first description of the movements of the swordfish occurring at high cool-temperate latitudes in the southwest Pacific demonstrates this subset of relatively large, likely mature fish has a high propensity for latitudinal mixing. Five of eleven swordfish crossed from the temperate Tasman Sea into tropical waters of the Coral Sea, up to ~ 3,400 km north of the point of capture into water temperatures of 29.0 °C.

### Partial migration between the Tasman and Coral Seas

The degree of latitudinal mixing demonstrated by some of the fish tagged in this study was notably greater than among those previously tagged off the east coast of Australia near the aforementioned tropical-temperate boundary, for which the extent of north–south movement was reported to be confined largely within 10° of latitude^8^. Of 30 swordfish tagged with PSATs between 23.9° and 29.4°S, only four were observed to move south of 35°S and one south of 40°S, despite similar lengths of PSAT deployments as the current study (43–364 days, median 131 days; see^68^). In the present study, while five swordfish (45%) predominantly lingered in the temperate Tasman Sea with movements spanning 2–6° of latitude, six (55%) migrated over 10.9–32.9° of latitude. While limited in sample size (n = 11), taken together with the findings of^8^, our results suggest a pattern of partial migration in the region, with a subset seasonally migrating between the Tasman Sea and Coral Sea, while other contingents remain regionally associated to either temperate or subtropical/tropical regions of the southwest Pacific.

Several factors probably contribute to maintain the observed pattern of partial latitudinal migration between the Coral Sea and the Tasman Sea. Occurrence in the Coral Sea, whether seasonal among the migrating contingent observed in this study, or year-round among regionally associated swordfish previously described^8^, likely reflects the distribution of habitat suitable for spawning, which is known to occur where sea surface temperature is at least 24 °C^69^. It has been postulated that swordfish caught and tagged in warmer waters may not need to migrate great latitudinal distances for reproduction as they are already in waters suitable for spawning^8^. By contrast, most swordfish tagged in this study were presumably reproductively mature based on their size^69,70^, and latitudinal migration from cool-temperate capture locations would be required for them to find spawning habitat.

Four temperate-to-tropical migrating fish in this study demonstrated periods of lingering behavior in warm, low latitude water after transit north, consistent with finding and using spawning habitat. Two of the migrating swordfish began low latitude lingering in September and October, respectively, coinciding with the start of the spawning season east of Australia (Sept. to Mar.;^69^). It is plausible that SC0024’s rapid transit to north of 30°S in the final week of deployment was also a spawning-related migration, as it occurred in the middle of the Dec.–Feb. peak of spawning season in the region^69^. Two other migrating fish began lingering in June and July, before this spawning season, but they remained in the Coral Sea after its putative start, until the end of their tag deployments in October. Worth noting is that these individuals also initially transited further north (11°S and 19°S, respectively) than the 25–30°S area where sampling was focused to establish a spawning season^69^. The spawning season of swordfish is probably more protracted in the tropics, as swordfish larvae occur north of 20°S in July–Sept. and year-round north of 10°S^71^. Thus, if the lingering behavior observed at low latitudes is spawning related, these results suggest swordfish off east Australia may have a broader spawning window than previously thought, facilitated by seasonal migration to suitable habitat.

Seasonal lingering in the Coral Sea occurred across a wide geographic area, so if the behavior is related to reproduction, this finding suggests spawning does not occur at a discrete spawning ground. Available evidence suggests spawning may instead track the transient distribution of suitable oceanographic habitat. It has been reported that a greater proportion of spawning condition swordfish occurring among those sampled in the East Australia Current (EAC) than among those sampled within close proximity in the same month, but just east of the warm influence of the EAC^69^. We found the latitudinal extent swordfish migrated into the Coral Sea was varied across the timing of the switch from transit to lingering: from ~ 10°S in austral winter (SC0007), ~ 18–19°S in spring and autumn (SC0004 and SC0008, respectively), and SC0012 to ~ 25°S in late spring/summer (the spawning season at that latitude;^69^). The switch from transit to lingering movement only occurred once water temperatures near the surface of 23–27 °C were encountered, suggesting the seasonal distribution of water warm enough for spawning may influence the extent of latitudinal seasonal migration.

The occurrence of swordfish in the temperate southwest Pacific is likely related to the presence of foraging habitat. Due to upwelling and downwelling eddies shed from the EAC into nutrient-rich temperate waters, the western Tasman Sea is highly productive and has the greatest non-coastal chlorophyll levels in the South Pacific^72,73^, providing a productive forage base. Relatively long latitudinal migrations of tracked swordfish into or both to-and-from cooler temperate waters have been reported from other regions globally, including the northwest and north Atlantic Ocean^19,74–76^, and the northwest^12^ and northeast Pacific^14,15^; as well as among other large predatory fish in the Pacific^77,78^. It is unclear whether the swordfish that were not observed to migrate out of the Tasman Sea in this study remain resident there, since all but one tag deployment ended prior to the Dec.–Feb. peak of spawning season^69^. If so, the pattern of high-latitude residency may indicate skipped spawning among a subset of the population^79^. The one tag still deployed that had not yet left the Tasman Sea through the peak spawning season (SC0014) instead encountered > 25 °C surface water in the EAC near the New South Wales continental shelf late in summer 2016–17. Therefore, variability in oceanographic features might occasionally obviate the need for migration out of the Tasman Sea to reach habitat suitable for spawning. As possible skipped spawning or temporary extensions of spawning habitat suggested by our limited sample may have implications for stock productivity, future investigation with longer tag deployments would be worthwhile.

While PSATs released from most of the migrating swordfish while they were still in the Coral Sea during the spawning season, SC0025 transited back to the Tasman Sea and was within 120 km of its capture location off southeast Tasmania when the tag released in February. This suggests the possibility of local site fidelity within the Tasman Sea. Site fidelity among large swordfish has been documented in the north Pacific^12,22^ and also from the northwest Atlantic, where swordfish undertook seasonal cool-temperate to tropical migrations beginning in autumn and returned to close vicinity of their capture location by the following summer^19^, a pattern also supported by conventional tag recaptures^80^. A propensity for local site fidelity, especially if in association with discernible bathymetric features is likely to increase susceptibility of swordfish to localized depletion^9,23^ and as such has been identified as a concern in the southwest Pacific that may warrant sub-regional management^81^. While the number of fish currently being caught by the southeast Australia recreational fishery is unlikely to have significant impact on the southwest Pacific swordfish stock overall, the fishery is confined to the narrow band of continental shelf break. In light of evidence of local site fidelity to this area, the recreational fishery would likely be affected by a local decline in abundance since the capacity to relocate fishing activity is inherently limited. The historical trajectories of other recreational swordfish fisheries demonstrate that participation is highly sensitive to perceived changes in catch rates^20^, and the potential for localized depletion will become an increasing concern if the number of fishers successfully engaging in the fishery expands in the future or if commercial catch in the area increases.

### Evidence of regional connectivity

The tag data presented here appears to support the hypothesis of limited connectivity of swordfish between the Tasman and Coral Seas and South Pacific to the east, as fish largely remained in the Tasman/Coral Sea basin for the duration of tag deployments. However, two of the 11 tracked fish were observed moving east of 165°E (one detected ~ 173°E before PSAT release), nominally a greater proportion than previously documented among swordfish tagged with PSATs near Australia (3 of 30;^8^). While sample size is limited, it is worth noting the two fish in this study that moved east of 165°E were relatively large (est. mass 140 and 280 kg) while the two that moved furthest east in the prior lower latitude Australian study were both 50 kg (est. dressed mass), the smallest of 30 tagged^68^, so these movements may reflect different underlying processes related to latitude between the two cohorts. The 165°E line of longitude separates regions used for the southwest Pacific stock assessment, and a greater relative degree of movement from the Tasman/Coral Sea into the South Pacific region to the east was noted to result in a more pessimistic stock status estimate^18^. As such, further tagging work including fish caught at high latitudes in the Tasman Sea may be useful to elucidate the extent of longitudinal movements of swordfish in the region.

### Diel vertical migration

Swordfish in this study overwhelmingly exhibited a pattern of diel vertical migration, descending during morning twilight into the mesopelagic zone typically to below 500 m depth, followed by an ascent back into the epipelagic zone during dusk. While day and night modal vertical distributions vary regionally, diel vertical migration into the mesopelagic zone has been reported fairly consistently across swordfish movement studies^8,13,14,24–27,29,74,75^. It has been suggested swordfish diel vertical migration is structured such that it follows an isolume or narrow band of light levels^15,25^, and as the main covariates of vertical movements identified are linked to light availability, our results appear to support a relationship of swordfish vertical movements and ambient illuminance.

### Moon phase influences nocturnal vertical movement behavior due to illuminance

The strongest predictor of night-time depth was moon phase, which has been identified previously to influence tagged swordfish vertical position^13,14,25^, perhaps due to its influence on swordfish prey night-time depth^32^. While we similarly demonstrate aggregated night-time depth increased around the full moon, by modelling tag depth at the finer temporal scale, we present evidence that this effect is notably variable across time of day — minimal near dusk and dawn and most pronounced between them. Since during the full moon, moonrise and moonset coincide with dusk and dawn and lunar downwelling illuminance is greatest between the two, this finding is consistent with illumination as the mechanism ultimately responsible. Similarly, we demonstrate that swordfish are also lower in the water column during near-full (i.e., gibbous) moon phases, but the effect is only present for the portion of the night that falls between moonrise and moonset. Lunar brightness increases exponentially with illuminated fraction (i.e., fullness), but the lunar contribution to downwelling luminance is similar across near-full moon phases as the peak elevation during full moon phases is lower in the sky^82^. As the proportion of night-time during which the moon’s elevation is > 0° (i.e., above the horizon) is positively correlated to illuminated fraction, it follows that a non-linear effect of moon phase on median swordfish night-time depth that gets more pronounced near the full moon should be expected assuming lunar illumination was responsible, which was observed in our night-time depth model. A similar pattern of daily aggregate night-time depth distributions being indistinguishable across moon phases up to ½–¾ full and then a pronounced increase in depth near the full moon was also documented among scattering layer organisms^83^ that swordfish are known to prey upon, likely due to the aforementioned mechanism of correlation between duration of night-time visible moon and illuminated fraction. As such, these results illustrate the importance of accounting for the unsteady relationship of moon phase and nocturnal downwelling illuminance, including across time of night, when investigating the moon’s effects on marine animals^82,84^.

The dynamics of moon phase on vertical distribution across time of night identified here also have implications for understanding and predicting fisheries dynamics. For example, several fishing strategies or ‘métiers’ used among Australian vessels targeting swordfish in the Coral/Tasman Seas with overnight longline sets have previously been identified^85^. Longlines with 12–13 hooks per float (approx. 20–120 m depth; see^41^) are deployed later in the evening in métiers used on waxing moon phases (i.e., when the moon is up before/at sunset), and earlier in the afternoon in a métier employed predominantly during waning phases after the full moon when moonrise occurs during the night (see Figs. S7–S9 of^85^). Thus, use of these métiers by fishers appears to optimize gear deployment timing for presentation during moonrise/set, which is when our results suggest swordfish shift vertically through the epipelagic with the rapid change in nocturnal illumination, perhaps maximizing the chance of swordfish interacting with hooks across the depth range of the longline catenary^41^. In regions where bycatch is a concern, characterizing the vertical dynamics of target species along with those of co-occurring species and fishing gear across moon phase and time of night would be particularly useful to inform targeted mitigation strategies.

### Daytime vertical movement behavior and mesopelagic habitat use

Water column light attenuation (K490) was the most important driver of swordfish daytime depth identified, with median daytime vertical position of swordfish rising ~ 195 m across the K490 values observed and plateauing toward high values. A similar relationship was described in^14^ and is consistent with swordfish maintaining low ambient illuminance by descending deeper in less turbid habitat. The greatest monthly K490 values were observed in the Tasman Sea, especially in spring during seasonal phytoplankton blooms.

Notably, we did not find evidence that temperature at 545 m depth influenced swordfish vertical habitat use during the day, suggesting routine mesopelagic habitat use of swordfish may not be constrained by low temperature. Despite the low temperatures experienced by swordfish in the mesopelagic, previous studies also inferred a lack of thermal limitation at depth^8,14,15^, perhaps because swordfish have a suite of functional adaptations for success in the mesopelagic zone. Large eyes^86^ with a uniquely adapted muscle that warms the brain and eyes^87^ up to 15 °C above ambient water temperature^27^ likely facilitate predatory success in dark cold water by providing superior visual acuity^88^. Unique cardiorespiratory adaptations^89–91^ appear to maintain function at low oxygen concentrations and across rapid large temperature changes experienced during vertical migration (e.g., swings of up to ∆ 22 °C between day and subsequent night in this study). Some previous tracking studies suggest observed surface basking behavior functions to ameliorate thermal debt that accrues while at depth or speed digestion^13,14,29^, because at equilibrium, swordfish only appear to be able to maintain muscle temperatures of ~ 1 °C above ambient^27,28^. In this study, however, while swordfish occasionally entered the top 50 m of the water column during the day, they only exhibited potential surface basking behavior (PSAT depth ≤ 3 m;^29^) on less than 2% of days on which shallow forays were recorded. The relationship of basking with environmental conditions is complex and is likely biotically mediated, for example by foraging success^14,29^, and the reason for its dearth in the present study is unclear. Whether the daytime forays into warm epipelagic water documented here provide the same proposed thermoregulatory role of surface basking documented in other regions or instead represent other functional behaviors (e.g., foraging^26^, reproduction^92^) is an area worth future investigation.

When in shallow epipelagic waters, swordfish maintain body temperatures similar to, or slightly above ambient, as they have a limited capacity for heterothermy outside of the head^28^. Despite this, during dives into the cooler mesopelagic they can retain enough residual heat that body temperature remains elevated above ambient after several hours at depth^28^. It follows that near-surface temperature may influence vertical habitat use in the cooler mesopelagic by determining how much residual heat the fish carry to depth, and we did find evidence that swordfish median daytime depth increased with sea surface temperature. However if this was a biologically-mediated causal relationship, we would expect the temperature of mesopelagic habitat (i.e., temp_545_) to also have an effect on daytime vertical habitat use, since the rate of residual heat loss at a given depth would be dependent on ambient temperature. As we did not find evidence of such an effect, we suggest sea surface temperature and similarly sea surface height may not be directly influencing swordfish use of mesopelagic habitat, and their modelled effects on median daytime depth may instead arise from latent correlation with unmodelled habitat covariates. For example, sea surface height and temperature are elevated in mesoscale anticyclonic eddies shed by the EAC^49,93^. It has been hypothesized that swordfish may either preferentially use or have altered vertical distributions within these warm-core eddies, as swordfish catch rates within them are sometimes elevated^94–96^. Other oceanographic features that correlate with surface covariate anomalies, like fronts, have similarly been shown to produce high catch rates^97^. Analyzing swordfish movement behavior in the context of potential preference for and use of oceanographic features could enhance accuracy and precision of fisheries dynamics models such as forecasting and catch rate standardization (e.g.,^98,99^). Although we were able to sample 1861 swordfish-days with high coverage of some covariates (e.g., moon phase), the correlated nature of conditions in the pelagic environmental across our 11 PSAT deployments likely limited the degree to which drivers of swordfish behavior could be isolated. Additional sampling to improve coverage of spatial and temporal variability in the southwest Pacific (especially at low latitudes and in summer/early autumn) would likely allow stronger inferences about swordfish habitat use to be drawn.

### What drives diel vertical migration of swordfish?

A critical knowledge gap in the ecology of swordfish as well as other large diel vertical migrating pelagic predatory fishes concerns the apparent relationship between ambient light levels and vertical migration. It is broadly assumed that rather than light being directly causal, the association arises from bottom-up pressure as predators follow the migration of mid-trophic level prey in the deep scattering layer^100–102^, which in turn vertically migrate in response to light^84^ or more complex trophic interactions^103^. Occasional swordfish deviations from constant-isolume diel vertical migration observed in some regions could also be explained by foraging-mediated vertical habitat selection, e.g., due to epipelagic prey availability or oxygen limitation of deep scattering layer depth^22,26^. With few exceptions however (e.g. ^104,105^), the hypothesis that diel vertical migration is driven by prey availability has surprisingly not been tested directly^102^.

While swordfish are broadly considered opportunistic predators, gut content analyses suggest non-random selection for prey that varies with ontogeny and location^101,106,107^. In the southwest Pacific, the taxonomic composition of and the proportion of organisms in the deep scattering layer that vertically migrate varies with depth through several hundred meters of the water column^108,109^. The presence of this complex, dynamically structured forage base along with evidence of swordfish prey selectivity suggests considerable scope for variation in a foraging-mediated model of swordfish vertical migration. Further, theoretical work on diel vertical migrating predator–prey interactions suggests individual variation in predatory strategies may be expected^110^. The presence of stable individual variation in swordfish behavior or ‘personality’ that influences trophic interactions in the mesopelagic would have ecological and evolutionary implications^111^, and the consistent differences in diel migration timing among two swordfish in this study may be preliminary evidence of such individual variation. Other theoretical predictions suggest the vertical position of predators may be driven by a trade-off between both proximity to prey and light availability at depth^112^. It follows that investigating swordfish movements in relation to both environmental conditions like illuminance and prey distribution in situ would be invaluable to understand their behavioral ecology and trophic interactions and degree of individual specialization therein, which has implications for understanding fisheries dynamics^75^ and critical ecosystem functions like carbon export^113^ in a changing ocean.

## Conclusions

The recent emergence of a recreational swordfish fishery near the species’ poleward range limit in the southwest Pacific has enabled a novel investigation of a high latitude subset of the population. These fish demonstrated a previously undocumented degree of connectivity between the temperate and tropical regions of the southwest Pacific, potentially to locate suitably warm spawning habitat. The sole migrating swordfish to still have a tag attached toward the end of the putative spawning season returned to within 120 km of its capture location, suggesting seasonal site fidelity. Two swordfish moved east of the 165°E parallel management boundary toward New Zealand, indicating a low but possibly greater degree of longitudinal connectivity across this management boundary than previously observed in the region. Variability in diel vertical migration into the mesopelagic zone during the day was related to light availability through factors like water column turbidity and moon phase, and the influence of the latter varies dynamically across time of night with implications for fisheries interactions. The aspects of movement behavior documented here are likely to influence reproduction, regional connectivity, and both trophic and fisheries interactions, important considerations for our understanding of this unique epi- and mesopelagic predator.

## Supplementary Information

Supplementary Information.

## Data Availability

The datasets generated during the current study are available from the corresponding author on reasonable request.
