# Supplementary material for: Movement behavior of swordfish provisions connectivity between the temperate and tropical southwest Pacific Ocean
Source: Sci Rep. 2023 Jul 21;13:11812. doi: 10.1038/s41598-023-38744-z (PMC10362066; doi:10.1038/s41598-023-38744-z)
Supplement: Supplementary file 1 — Supplementary Information. [file 41598_2023_38744_MOESM1_ESM.docx]

**Supplementary Information for:** Movement behaviour of a top pelagic predator provisions connectivity between the temperate and tropical southwest Pacific Ocean

Sean R. Tracey^1^, Barrett Wolfe^1^, Klaas Hartmann^1^, Julian Pepperell^2^, Sam M. Williams^3^

This file contains Supplementary Tables S1–S5 and Supplementary Figures S1–S4.

Supplementary Table S1. Hidden Markov Model parameters dispersion and state switching probabilities

| ID | σ_1_ | σ_2_ | P_11_ | p_22_ |
| --- | --- | --- | --- | --- |
| SC0004 | 1.595 | 0.36 | 0.879 | 0.931 |
| SC0008 | 1.104 | 0.231 | 0.795 | 0.914 |
| SC0014 | 1.747 | 0.602 | 0.098 | 0.066 |
| SC0016 | 1.744 | 0.578 | 0.745 | 0.864 |
| SC0013 | 1.57 | 0.503 | 0.219 | 0.846 |
| SC0012 | 1.729 | 0.452 | 0.903 | 0.91 |
| SC0007 | 1.612 | 0.671 | 0.837 | 0.902 |
| SC0019 | 1.708 | 0.553 | 0.05 | 0.867 |
| SC0024 | 2.55 | 0.531 | 0.731 | 0.938 |
| SC0023 | 1.728 | 0.42 | 0.481 | 0.8 |

Supplementary Table S2. Total length of PSAT data series after removing spurious data at the beginning and end of deployments, and percent recovery of temperature and depth data which are sampled from PSAT sensors every ten minutes.

| **ID** | **Days** | **Temp.**  **recovered** | **Depth**  **recovered** |
| --- | --- | --- | --- |
| SC0004 | 249.2 | 64.2% | 64.2% |
| SC0007 | 185.2 | 58.6% | 61.9% |
| SC0008 | 183.8 | 83% | 82.7% |
| SC0012 | 250.3 | 60.4% | 62.9% |
| SC0013 | 160.2 | 62.6% | 63.5% |
| SC0014 | 250.5 | 75.3% | 75.2% |
| SC0016 | 126.7 | 75.1% | 70.9% |
| SC0019 | 55.0 | 96.8% | 96.4% |
| SC0023 | 77.0 | 85.3% | 84.4% |
| SC0024 | 214.9 | 67.7% | 71.5% |
| SC0025 | 77.1 | 72.2% | 71.0% |
|  |  |  |  |

Supplementary Table S3. Diel diving behaviour log-link gamma general additive model summary. Smooth functions are in notation of R package *mgcv*. See manuscript ‘Methods’ for details.

| **Component** | **Term** | **Estimate** | **Std Error** | **t-value** | **p-value** |
| --- | --- | --- | --- | --- | --- |
| A. parametric coefficients | (Intercept) | 5.167 | 0.085 | 60.598 | <0.0001 |
|  |  |  |  |  |  |
| **Component** | **Term** | **edf** | **Ref. df** | **F-value** |  |
| B. smooth terms | s(time) | 35.142 | 46.000 | 107.345 | <0.0001 |
|  | s(DoY) | 20.300 | 22.000 | 21.590 | <0.0001 |
|  | s(moon) | 11.788 | 18.000 | 33.871 | <0.0001 |
|  | s(lat) | 18.751 | 22.525 | 5.123 | <0.0001 |
|  | s(DoY):SC0019 | 0.005 | 11.000 | 0.000 | 0.2846 |
|  | s(DoY):SC0024 | 15.452 | 19.000 | 33.619 | 0.0035 |
|  | s(DoY):SC0023 | 6.345 | 9.000 | 32.672 | 0.1405 |
|  | s(DoY):SC0025 | 0.003 | 14.000 | 0.000 | 0.5999 |
|  | s(DoY):SC0004 | 16.085 | 21.000 | 8.205 | 0.0001 |
|  | s(DoY):SC0016 | 3.646 | 13.000 | 9.039 | 0.1265 |
|  | s(DoY):SC0007 | 13.047 | 17.000 | 7.943 | <0.0001 |
|  | s(DoY):SC0014 | 12.909 | 21.000 | 14.210 | <0.0001 |
|  | s(DoY):SC0013 | 7.046 | 16.000 | 15.799 | 0.0002 |
|  | s(DoY):SC0008 | 12.795 | 17.000 | 4.617 | 0.0002 |
|  | s(DoY):SC0012 | 4.774 | 22.000 | 7.173 | <0.0001 |
|  | s(time):SC0019 | 4.961 | 46.000 | 0.345 | 0.0013 |
|  | s(time):SC0024 | 19.170 | 46.000 | 20.189 | <0.0001 |
|  | s(time):SC0023 | 5.744 | 46.000 | 0.993 | <0.0001 |
|  | s(time):SC0025 | 3.989 | 46.000 | 0.409 | 0.0001 |
|  | s(time):SC0004 | 13.937 | 46.000 | 1.015 | <0.0001 |
|  | s(time):SC0016 | 28.746 | 46.000 | 3.186 | <0.0001 |
|  | s(time):SC0007 | 31.498 | 46.000 | 7.693 | <0.0001 |
|  | s(time):SC0014 | 5.364 | 46.000 | 0.893 | <0.0001 |
|  | s(time):SC0013 | 10.985 | 46.000 | 4.580 | <0.0001 |
|  | s(time):SC0008 | 33.406 | 46.000 | 5.180 | <0.0001 |
|  | s(time):SC0012 | 0.002 | 46.000 | 0.000 | 0.0971 |
|  | s(SC-FishID) | 4.773 | 10.000 | 1.879 | <0.0001 |
|  | ti(DoY,time) | 437.557 | 1,058.000 | 3.469 | <0.0001 |
|  | ti(moon,time) | 184.083 | 1,288.000 | 2.995 | <0.0001 |
|  | ti(lat,time) | 153.616 | 1,104.000 | 0.591 | <0.0001 |
|  | ti(lat,DoY,time) | 819.538 | 1,932.000 | 2.399 | <0.0001 |
| Adjusted R-squared: 0.888, Deviance explained 0.836, Scale est: 0.308, N: 185809 | | | | | |
|  | | | | | |

Supplementary Table S4. Median daily daytime depth general additive model summary.

| **Component** | | **Term** | **Estimate** | **Std Error** |  | **t-value** | **p-value** |  |
| --- | --- | --- | --- | --- | --- | --- | --- | --- |
| A. parametric coefficients | | (Intercept) | -492.562 | 18.564 |  | -26.534 | <0.0001 |  |
| **Component** | |  | **edf** | **Ref. df** |  | **F-value** | |  |
| B. smooth terms | | s(temp2.5) | 2.771 | 9.000 |  | 4.392 | <0.0001 |  |
|  |  | s(SSH) | 0.624 | 9.000 |  | 0.208 | 0.0882 |  |
|  |  | s(K490) | 2.714 | 9.000 |  | 7.984 | <0.0001 |  |
|  |  | s(SC-FishID) | 7.784 | 10.000 |  | 6.179 | <0.0001 |  |
|  |  | s(temp2.5):SC0019 | 0.000 | 8.000 |  | 0.000 | 0.4478 |  |
|  |  | s(temp2.5):SC0024 | 0.000 | 8.000 |  | 0.000 | 0.4760 |  |
|  |  | s(temp2.5):SC0023 | 0.000 | 8.000 |  | 0.000 | 0.4751 |  |
|  |  | s(temp2.5):SC0025 | 0.000 | 8.000 |  | 0.000 | 0.9402 |  |
|  |  | s(temp2.5):SC0004 | 1.820 | 8.000 |  | 0.536 | 0.0257 |  |
|  |  | s(temp2.5):SC0016 | 2.496 | 8.000 |  | 1.598 | 0.0003 |  |
|  |  | s(temp2.5):SC0007 | 2.220 | 8.000 |  | 0.783 | 0.0088 |  |
|  |  | s(temp2.5):SC0014 | 1.810 | 8.000 |  | 0.315 | 0.2128 |  |
|  |  | s(temp2.5):SC0013 | 2.760 | 8.000 |  | 1.990 | 0.0007 |  |
|  |  | s(temp2.5):SC0008 | 0.000 | 8.000 |  | 0.000 | 0.9957 |  |
|  |  | s(temp2.5):SC0012 | 4.648 | 8.000 |  | 3.054 | 0.0002 |  |
|  |  | s(SSH):SC0019 | 0.849 | 8.000 |  | 0.170 | 0.1644 |  |
|  |  | s(SSH):SC0024 | 0.000 | 8.000 |  | 0.000 | 0.5480 |  |
|  |  | s(SSH):SC0023 | 0.000 | 8.000 |  | 0.000 | 0.9244 |  |
|  |  | s(SSH):SC0025 | 0.000 | 8.000 |  | 0.000 | 0.9861 |  |
|  |  | s(SSH):SC0004 | 0.000 | 8.000 |  | 0.000 | 0.4313 |  |
|  |  | s(SSH):SC0016 | 3.231 | 8.000 |  | 1.626 | 0.0013 |  |
|  |  | s(SSH):SC0007 | 6.136 | 8.000 |  | 2.555 | 0.0006 |  |
|  |  | s(SSH):SC0014 | 0.000 | 8.000 |  | 0.000 | 0.7114 |  |
|  |  | s(SSH):SC0013 | 0.000 | 8.000 |  | 0.000 | 0.4204 |  |
|  |  | s(SSH):SC0008 | 0.000 | 8.000 |  | 0.000 | 0.9983 |  |
|  |  | s(SSH):SC0012 | 0.000 | 8.000 |  | 0.000 | 0.9789 |  |
|  |  | s(K490):SC0019 | 3.069 | 8.000 |  | 5.934 | 0.0062 |  |
|  |  | s(K490):SC0024 | 4.606 | 8.000 |  | 5.883 | <0.0001 |  |
|  |  | s(K490):SC0023 | 2.373 | 8.000 |  | 1.390 | 0.0117 |  |
|  |  | s(K490):SC0025 | 0.000 | 8.000 |  | 0.000 | 0.8845 |  |
|  |  | s(K490):SC0004 | 2.345 | 8.000 |  | 0.794 | 0.0435 |  |
|  |  | s(K490):SC0016 | 3.666 | 8.000 |  | 3.195 | <0.0001 |  |
|  |  | s(K490):SC0007 | 3.697 | 8.000 |  | 1.727 | 0.0007 |  |
|  |  | s(K490):SC0014 | 0.357 | 8.000 |  | 0.050 | 0.2340 |  |
|  |  | s(K490):SC0013 | 5.241 | 8.000 |  | 7.108 | <0.0001 |  |
|  |  | s(K490):SC0008 | 0.000 | 8.000 |  | 0.000 | 0.7738 |  |
|  |  | s(K490):SC0012 | 0.000 | 8.000 |  | 0.000 | 0.4638 |  |
|  |  | s(lon,lat):SC0019 | 0.001 | 24.000 |  | 0.000 | 0.4333 |  |
|  |  | s(lon,lat):SC0024 | 5.924 | 51.000 |  | 0.162 | 0.0861 |  |
|  |  | s(lon,lat):SC0023 | 16.417 | 40.000 |  | 1.345 | <0.0001 |  |
|  |  | s(lon,lat):SC0025 | 0.000 | 13.000 |  | 0.000 | 0.6547 |  |
|  |  | s(lon,lat):SC0004 | 0.000 | 15.000 |  | 0.000 | 0.8215 |  |
|  |  | s(lon,lat):SC0016 | 0.001 | 31.000 |  | 0.000 | 0.4169 |  |
|  |  | s(lon,lat):SC0007 | 0.000 | 7.000 |  | 0.000 | 0.3810 |  |
|  |  | s(lon,lat):SC0014 | 6.302 | 30.000 |  | 0.409 | 0.0193 |  |
|  |  | s(lon,lat):SC0013 | 6.243 | 45.000 |  | 0.235 | 0.0449 |  |
|  |  | s(lon,lat):SC0008 | 0.000 | 12.000 |  | 0.000 | 0.9982 |  |
|  |  | s(lon,lat):SC0012 | 1.605 | 40.000 |  | 0.047 | 0.2729 |  |
|  | Adjusted R^2^: 0.625, Deviance explained 0.650, Scale est: 5417.1, N: 1484 | | | | | | | |

|  |
| --- |

Supplementary Table S5. Median daily night-time log(depth) general additive model summary

| **Component** | **Term** | **Estimate** | **Std Error** | **t-value** | **p-value** |
| --- | --- | --- | --- | --- | --- |
| A. parametric coefficients | (Intercept) | 3.274 | 0.141 | 23.272 | <0.0001 |
| **Component** | **Term** | **edf** | **Ref. df** | **F-value** |  |
| B. smooth terms | s(moon) | 7.607 | 18.000 | 7.168 | <0.0001 |
|  | s(SC-FishID) | 9.512 | 10.000 | 26.299 | <0.0001 |
|  | s(moon):SC0019 | 0.000 | 8.000 | 0.000 | 0.8756 |
|  | s(moon):SC0024 | 0.000 | 8.000 | 0.000 | 0.7887 |
|  | s(moon):SC0023 | 0.000 | 8.000 | 0.000 | 0.7936 |
|  | s(moon):SC0025 | 0.000 | 8.000 | 0.000 | 0.9928 |
|  | s(moon):SC0004 | 0.676 | 8.000 | 0.113 | 0.2374 |
|  | s(moon):SC0016 | 0.569 | 8.000 | 0.095 | 0.2470 |
|  | s(moon):SC0007 | 1.711 | 8.000 | 0.613 | 0.0378 |
|  | s(moon):SC0014 | 0.000 | 8.000 | 0.000 | 0.7985 |
|  | s(moon):SC0013 | 1.662 | 8.000 | 0.526 | 0.0468 |
|  | s(moon):SC0008 | 1.334 | 8.000 | 0.357 | 0.0879 |
|  | s(moon):SC0012 | 0.715 | 8.000 | 0.128 | 0.2104 |
|  | s(lon,lat):SC0019 | 0.000 | 18.000 | 0.000 | 0.9839 |
|  | s(lon,lat):SC0024 | 11.117 | 40.000 | 0.542 | 0.0117 |
|  | s(lon,lat):SC0023 | 0.000 | 31.000 | 0.000 | 0.6383 |
|  | s(lon,lat):SC0025 | 0.000 | 9.000 | 0.000 | 0.9958 |
|  | s(lon,lat):SC0004 | 0.000 | 16.000 | 0.000 | 0.4681 |
|  | s(lon,lat):SC0016 | 0.000 | 26.000 | 0.000 | 0.4376 |
|  | s(lon,lat):SC0007 | 0.000 | 8.000 | 0.000 | 0.9001 |
|  | s(lon,lat):SC0014 | 3.502 | 26.000 | 0.209 | 0.1288 |
|  | s(lon,lat):SC0013 | 2.309 | 39.000 | 0.071 | 0.2699 |
|  | s(lon,lat):SC0008 | 0.000 | 11.000 | 0.000 | 0.7289 |
|  | s(lon,lat):SC0012 | 5.435 | 34.000 | 0.303 | 0.0281 |
| Adjusted R^2^: 0.462, Deviance explained 0.478, Scale est: 0.395, N: 1566 | | | | | |


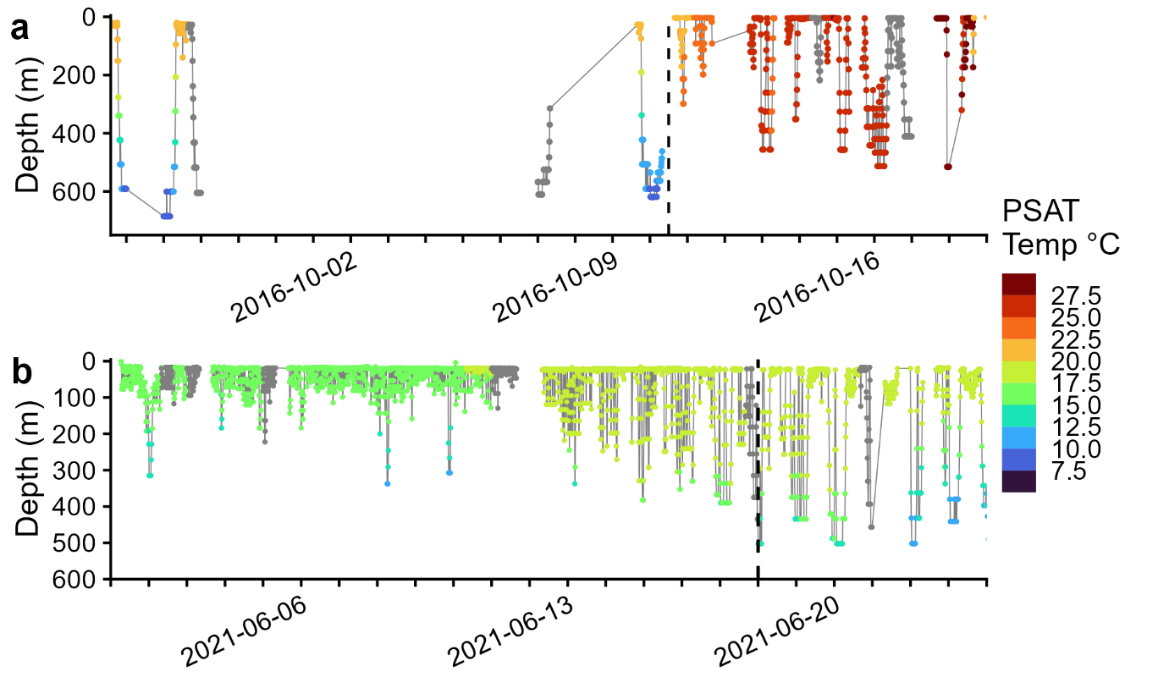


Supplementary Figure S1. PSAT data removed from analyses. In the final week before the PSAT reported, SC0007 (**a**) depth patterns became erratic and recorded temperature was sSupplementary Table from the surface to 400+ m depth, suggesting consumption by a heterothermic predator such as a lamnid shark (e.g., shortfin mako *Isurus oxyrinchus* or white *Carcharodon carcharias*). Data recorded after the dashed line were omitted from all analyses. Note that the large gap was a period for which data is missing but typical daytime diving behaviour and water temperatures at depth were observed just prior to the end of the series SC0023 (**b**) displayed an extended period of abnormal diving behaviour during the first two weeks of deployment, remaining almost entirely within the top 100 m of the water column. Subsequently the swordfish gradually returned to a more pronounced diel vertical migration and several additional days (18 total) were conservatively removed, assuming this was representative of recovery from stress induced during capture with recreational angling gear.


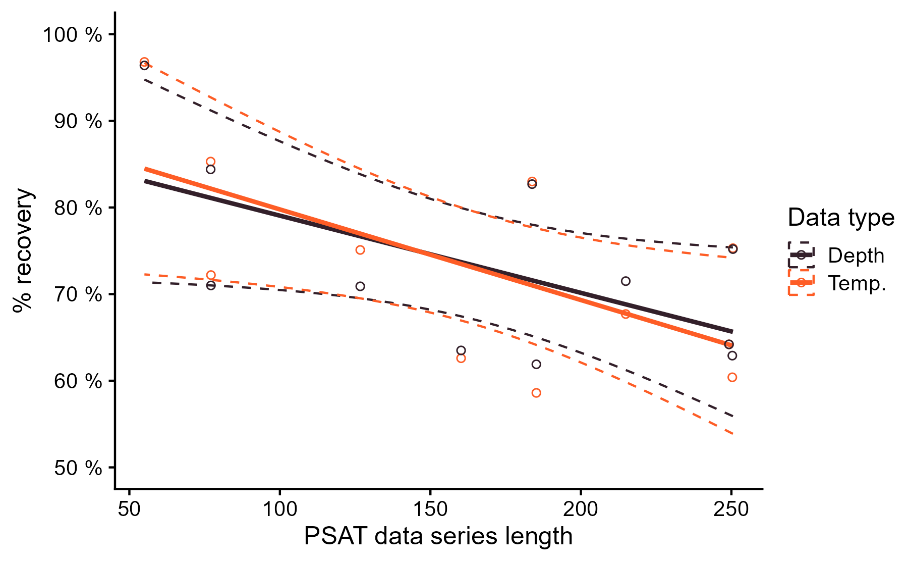


Supplementary Figure S2. Proportion of PSAT data series recovery through the ARGOS system versus length of PSAT deployments.


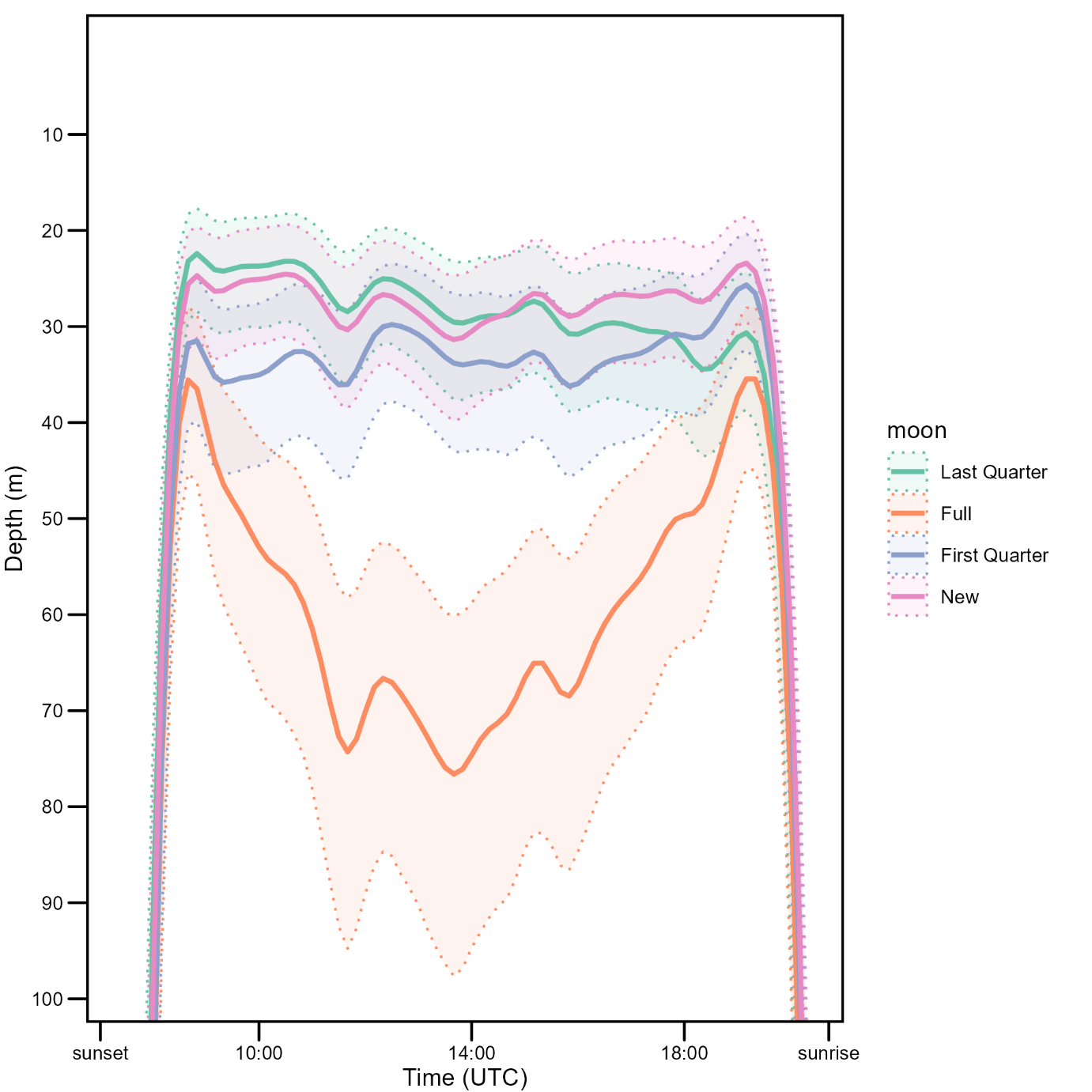


Supplementary Figure S3. Depth profiles across moon phase predicted by the vertical behaviour model (GAM). Shaded bands are credible intervals. Last and First Quarter phases are the waning and waxing half-full phases, respectively.


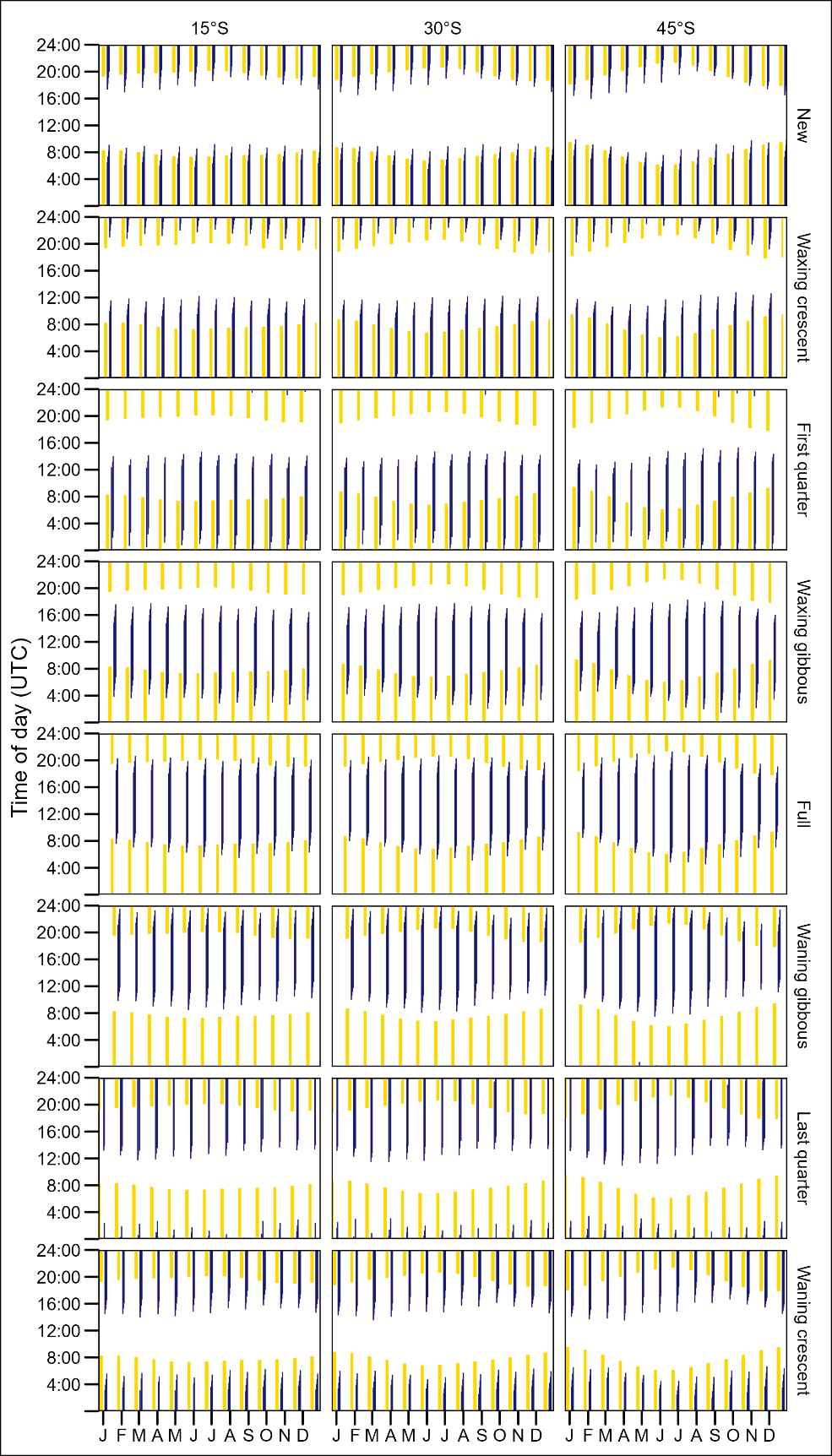


Supplementary Figure S4. Timing of moon visibility (moon elevation > 0° i.e., above the horizon) relative to daytime across the year, by latitude and moon phase, at different latitudes across the southwest Pacific at 155°E longitude. Yellow bars indicate daytime (between sunrise and sunset), dark blue bars (staggered from corresponding daytimes) indicate the period between moonrise and moonset at a given latitude. Moon and sun times calculated with the R package *suncalc*.


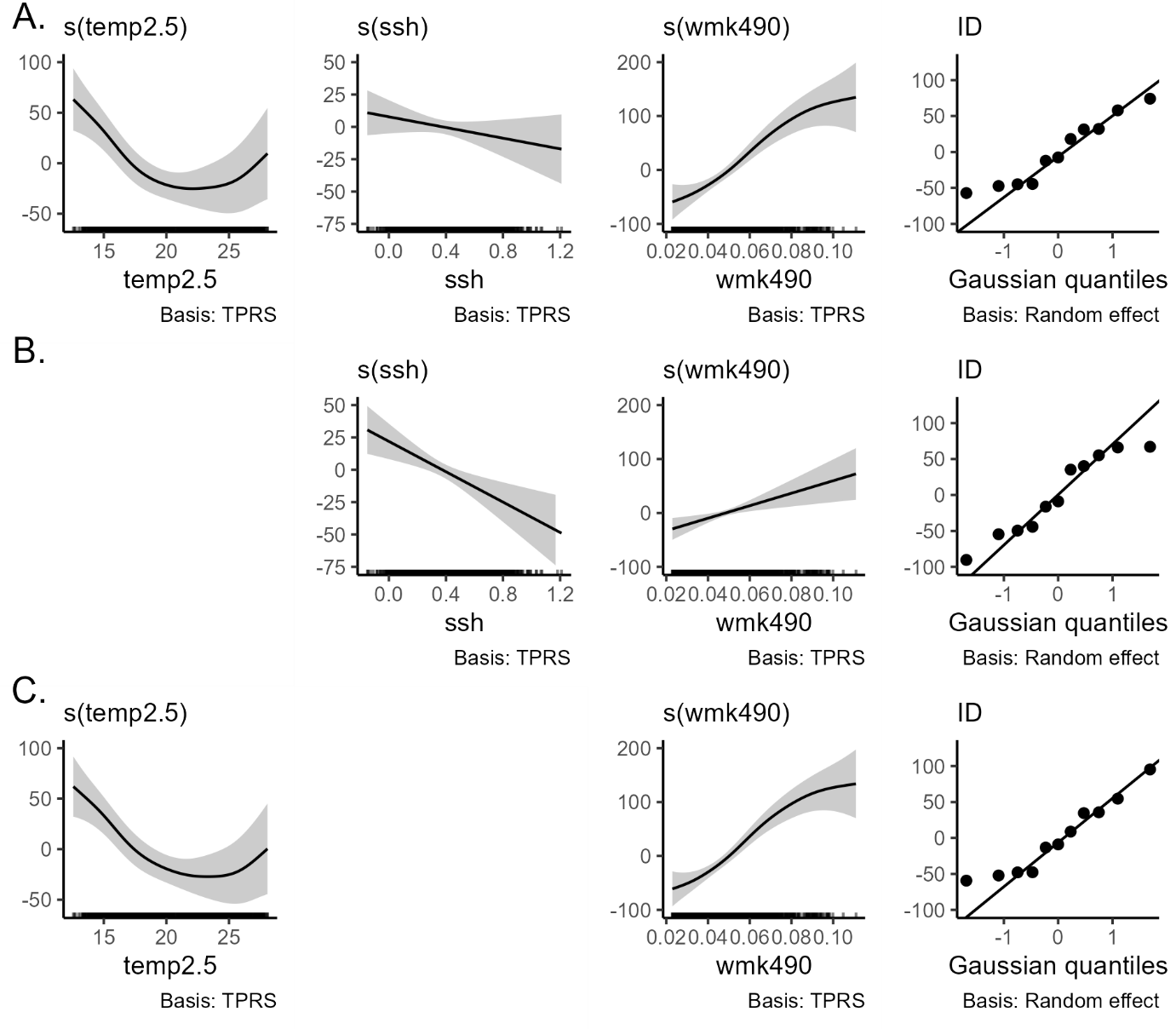


Supplementary Figure S5. Partial effects of smooth terms of the median daytime depth model (A), the model with the temp2.5 (i.e., sea surface temperature) term omitted (B), and the model with the ssh (sea surface height) term omitted (C). While s(ssh) was moderately concurve on s(temp2.5) (Figure 9), removing the term does not notably alter the effects of other terms.
